# Sustainable, Renewable and Environmental-Friendly Insulation Systems for High Voltages Applications

**DOI:** 10.3390/molecules25173901

**Published:** 2020-08-27

**Authors:** Muhammad Rafiq, Muhammad Shafique, Anam Azam, Muhammad Ateeq, Israr Ahmad Khan, Abid Hussain

**Affiliations:** 1 Department of Electrical Engineering, University of Engineering & Technology, Taxila 47050, Pakistan; israr@uettaxila.edu.pk; 2 Department of Architecture and Civil Engineering, City University of Hong Kong, Hong Kong 999077, China; 3 School of Economics and Management, North China Electric Power University, Beijing 102206, China; anamrafiq95@gmail.com; 4 Department of Mechanical Engineering, COMSATS University Islamabad, Sahiwal Campus, Sahiwal 57000, Pakistan; ateeqmuhammad93@gmail.com; 5 Department of Mechanical Engineering, University of Engineering & Technology, Taxila 47050, Pakistan; abid.hussain@uettaxila.edu.pk

**Keywords:** renewable insulation, sustainable oil, environmentally friendly, vegetable oil, natural ester, transformer insulation

## Abstract

With the inception of high voltage (HV), requisites on the insulating permanence of HV equipment is becoming increasingly crucial. Mineral/synthetic oil liquid insulation—together with solid insulation materials (paper, pressboard)—is the fundamental insulation constituent in HV apparatuses; their insulation attributes perform a substantial part in a reliable and steady performance. Meanwhile, implications on the environment, scarcity of petroleum oil supplies and discarding complications with waste oil have stimulated investigators to steer their attention towards sustainable, renewable, biodegradable and environmentally friendly insulating substances. The contemporary insulating constituent’s evolution is driven by numerous dynamics—in particular, environmental obligations and other security and economic issues. Consequently, HV equipment manufacturers must address novel specifications concerning to these new standards. Renewable, sustainable and environmentally friendly insulating materials are continuously substituting conventional insulating items in the market place. These are favorable to traditional insulating materials, due to their superior functionality. The also offer explicit security and eco-friendly advantages. This article discusses cutting-edge technology of environmentally friendly insulating materials, including their fabrication, processing and characterization. The new renewable, insulating systems used in HV equipment are submitted and their fundamental gains stated in comparison with conventional insulating materials. Several experimental efforts carried out in various parts of the world are presented, offering an outline of the existing research conducted on renewable insulating systems. The significance of this article lies in summarizing prior investigations, classifying research essence, inducements and predicting forthcoming research trends. Furthermore, opportunities and constraints being experienced in the field of exploration are evidently reported. Last but not least, imminent research proposals and applications are recommended.

## 1. Introduction

The challenges related to environmental consequences of industrial operations will potentially endure in the future with increased population, need for engineering-related products and the economic [1]. Every industrial operation produces remains and discarded yields that may impact the atmosphere, particularly which are categorized as non-renewable and non-eco-friendly. The requirement of clean expertise and residue handling has surfaced as one of the most significant concerns of recent times, demanding explicit activities to inhibit debris production and stimulate recycle and reuse actions to decrease ecological impacts. Consequently, contemporary guidelines and “green” measures have seemed to inspire atmosphere security because of the rapid rise of disposals in the manufacturing field. Due to rising global energy demands, the electricity industry is confronting this problem. The electricity sector is constantly emerging since the commencement [2]. During the last few decades, technology innovations have surfaced to address imminent energy requirements, highlighting primarily two subjects: sustainability and efficiency [3]. The electric transmission effectiveness depends on the capability to decrease losses by decreasing transmitted current, which has been probable by the manufacturing of HV apparatus, e.g., bushings, capacitors and power transformers, etc. The grid of the future must consist of contemporary equipment with innovative design, modern insulating substances and novel expertise to assure safer, consistent, stable and green electricity [4].

Electricity is supposed to be a fundamental need of modern society and it is usually the backbone for economic development and welfare of society. The electrical grid is an electric power system consists of power-producing stations, transmission cables, substations, transformers and distribution lines. This network is mainly responsible for integrating cities and appreciates social activities combining with economic, public and environmental systems with population and urban development. In addition, the power system also contributes to socioeconomic growth and improves the standard of living through developing inter-to intra-city grids during expansion. The demand for development of a prospective low carbon grid has increased enormous stresses on the stability and efficiency of dielectric materials used in power systems to meet the impulsive and vivid working conditions [5]. The future electrical power system must possess the ability to cope with the prompt progression of power load and its asymmetric distribution demand for huge capacity, extended distance and low-loss power transmission. This kind of network interconnects the grids in various countries and even different continents to confirm a secure and consistent supply of energy.

High-voltage direct current (HVDC) electric transmission technology is an ultimate solution to enhance the capacity. For instance, ± 1100 kV DC enables to transmit at a distance of 5000 km and a volume of 12 GW, which adequately covers the requirements of intercontinental electric grid interconnection. In order to facilitate the aforementioned interconnections of networks in separate regions, future electric insulation systems must be capable of dealing with larger capacity and higher voltages [6]. The liquid insulation system is applied both as an insulator and a coolant in several elements of the HV network comprising cables, switchgear and transformers. Nevertheless, currently, mineral oil (MO) is being used in almost all of these applications as liquid insulation for almost a century which is detrimental to the environment and emanates from a non-renewable origin (crude oil) [7]. Meanwhile, as a consequence of the scarcity of oil reserves, sustainable development of oil is strongly discussed globally since it is gradually accepted that first-generation MO, mainly developed from petroleum derivatives, is limited in resources and also possess multiples detriments, e.g., non-biodegradability, non-renewable, lower flash point and also may lead to other serious issues if there is leakage. Moreover, huge automation and industrialization in the community have driven towards the enormous demand for petroleum derivatives. The aforementioned issues have sought huge focus from researchers to search for a substitute, which may be developed from substances accessible plentiful naturally and which probably could submit paramount prospects in the longer run.

**Figure 1:**
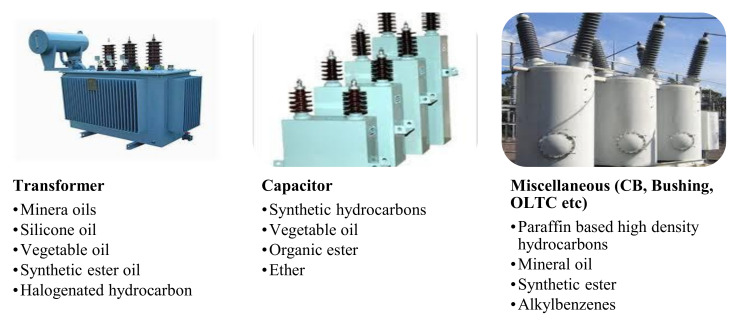
Classification of liquid insulating fluids based on their application.

Most of the HV equipment for example, transformers, capacitors, switches and circuit breakers, use billions of liters of insulating liquid (as shown in Figure 1). This oil basically performs three essential functions, i.e., (1) it electrically offers insulation between different active elements and also performs as a preventive coating film to avoid corrosion of metallic areas; (2) it efficiently removes the heat from core and conductors by the action of conduction and transports heat to enclosing container, that is later discharged outward to the environment and (3) it behaves as a healthiness index for HV equipment. Using condition monitoring analysis of oil, the state of electric equipment can be constantly monitored. It delivers diagnostic backing to evaluate the condition/healthiness of the apparatus through systematic monitoring. MO has been used as an insulating and cooling medium for almost a century in HV equipment. This equipment is sometimes used in environmentally sensitive regions for example, shopping malls and waterways. The leakage of oil in these areas may lead to fire or may contaminate the water and environment. Over the past few years, these environmental problems have been expressed on the application of badly biodegradable liquids in electric equipment in spaces where leaks and equipment collapse may lead to contamination of the environment. In comparison to contamination of land, contamination of waterways is more critical [8].

Environmentally friendly insulating materials have surfaced as a stimulating and innovative substitute to address and satisfy environmental and security challenges in numerous industrial segments—particularly in the electricity sector. Environment-friendly insulating liquids for HV apparatuses is an outstanding illustration of eco-matters for energy applications. For over a century, MO has been applied as dielectric fluid for common HV machinery owing to its outstanding physical, chemical, dielectric traits and it is the relatively lesser cost [9,10]. On the contrary, innovative “green” insulating liquids have lately emerged as a green alternative to substitute hydrocarbon-based derivatives as they not simply possess suitable features for application in HV apparatus, but also fulfill environment and health demands for instance nontoxicity, biodegradability, recyclability and non-hazardous, etc.; furthermore, they are classified as fire-resistant liquids [11]. Few of the environmental substitutes are ester-based liquids, synthetic and natural esters (NEs) [12].

Vegetable oils (VOs) are assumed to be an appropriate replacement of MO in high voltage equipment. They are extracted naturally from seeds, flowers or less likely from other segments of fruits. Numerous investigators and industries are conducting research to examine their effectiveness as insulating fluids in HV equipment [13]. VGs are highly biodegradable (˃95%), lesser toxic, lesser flammable and have an extraordinary flashpoint (˃300 °C) and fire points (˃300 °C). Furthermore, they are also more environmentally friendly liquids [14,15]. Additionally, these oils absorb more moisture in comparison to MOs [16]. Nevertheless, extraordinary loadings of unsaturated fatty acid transform them volatile and vulnerable to oxidation [17]. This fatty acid and its level of unsaturation impact the physiochemical and insulating traits of VOs. These VOs possess more acidity than MOs because of hydrolysis reaction which develops the aforementioned acids (which does not occur in MOs) and due to dissimilar chemical configuration of two oils [18]. Similarly, the character of confined acids in duo oils is disparate. VOs generally have great molecular acids (HMA) like oleic and stearic acids however, MOs comprise low molecular weight acids (LMA) such as formic, acetic and levulinic acids [19,20].

The research drive towards developing a wholly biodegradable liquid insulation commenced in the 1990s as a result of utility curiosity. Several R & D efforts started in this course to develop renewable fluid insulation. The VOs were deliberated the most prospective applicant for a totally biodegradable, sustainable, renewable and environmentally friendly liquid insulation. Undoubtedly, VOs are abundantly available as a natural resource and thought to be green and reasonable insulator [21]. VOs have evolved as a progressively common substitute of MOs. They generally offset most of the major hazards attached with common MOs, for example, extraordinary flammability and adverse ecological effects. These are developed from renewable organic resources in particular seeds, vegetables, flowers, etc. They are decomposable, nontoxic and hold small radiation outlines. Similarly, the application of VOs fluids is ecologically favorable.

During the last couple of decades, vegetable oils became accessible as liquid insulation for HV applications. The leading notable marketable item was BIOTEMP^®^, trademarked by ABB USA in September 1999 [22]. The base liquid was great oleic oil comprising of 80% oleic amount. This oil was developed from seeds that were prepared by elected reproduction; lately, controlling genetic practices had likewise been applied. Further partial hydrogenation phase was applied to curtail unstable tri-unsaturates. This BIOTEMP^®^ liquid insulation has been used in different HV equipment installed in certain sensitive regions. Later in the same year, one more US patent was delivered, for liquid insulation acquired from soybean oil presented by Waverly Light & Power [23]. During March 2000, Cooper industries presented oil insulation under the trademark Environtemp FR_3_^®^ [24]. This was developed from standard class oleic base oils and also applied in HV equipment. In August 2001, another patent was supplied by ABB on BIOTEMP^®^ [25].

This study provides a critical analysis of the recent research on Sustainable, renewable and environmental-friendly insulation for transformer and an extensive literature review can provide an essential concept of regarding the basic knowledge of insulation for transformer applications in high voltage design and indicate future research agenda. The following main steps were taken for the literature collection. The primary stage includes the search in Google Scholar and Scopus portals. In this search, the top engineering journals containing the “insulation for transformer” “green insulation” keywords in the title and keywords were selected. The keywords related to the above topic were also investigated and the related data were collected from several journals.

Second, main keywords, such as “Sustainable insulation,” “renewable insulation,” and “High voltage insulation,” were identified for our topic. Later, a combination of phrases was identified and selected based on the available scientific data and knowledge of the research group. As of 01 April 2020, titles, abstracts and keywords were searched in the above two databases. Based on the selected keywords search, a total of over 312 papers were collected. In addition, all the paper abstracts were screened out and documents out of scope were removed. Lastly, 176 which were related to our topic, were extensively studied and included in our review paper.

## 2. Dielectric Fluids HV Applications

One of the utmost significant constituents of HV equipment is the insulating liquid as it behaves both as electrical insulation and as a cooling media [26]. Every explicit application requires an insulating material with definite electrical, physical and chemical qualities, together with outstanding insulating strength, chemical and thermal stability, superior heat transfer features and in specific instances, nonflammability and smaller toxicity conduct [27].

Naturally, several elements of HV apparatus in the electric network for instance, transformers, capacitors, bushings, switches and circuit breakers, use insulating liquids for insulation and cooling. The insulation system generally applied in HV apparatus solid cellulose insulation (paper/pressboard) and liquid insulation for stable functioning.

**Figure 2:**
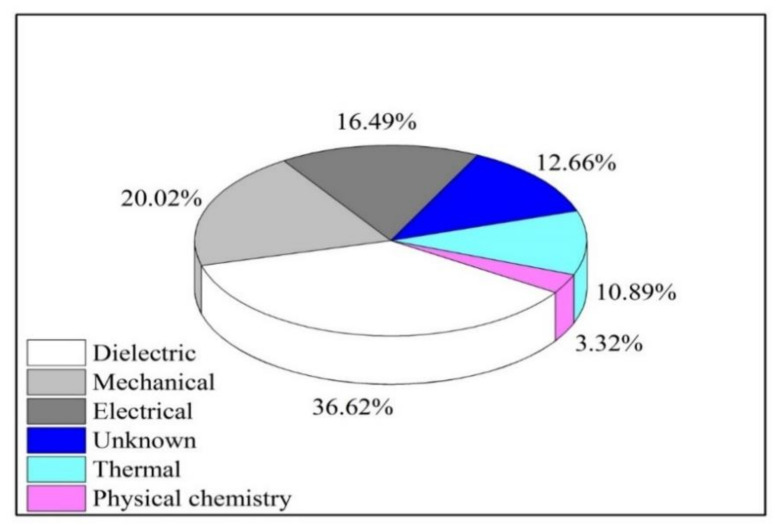
Main causes of high voltage (HV) equipment (especially transformer) collapse [29] with permission from Elsevier.

In the electricity sector, this is acknowledged that the lifespan of the HV apparatus is primarily based upon used insulation arrangement (usually liquid and solid insulating materials) status. This arrangement forms the main insulating constituent of the insulation system executing the threefold operation of the electric barrier, mechanical backing and heat outflow route. The statistics indicate most of the HV equipment failures are caused by insulation issues (as indicated in Figure 2). Consequently, a suitable choice and right preservation of insulating material are crucial to maintaining prolong its lifetime and to maintain operational reliability [28]. It is highly significant to recognize all the properties, primarily electrical, physical and thermal of the insulating material to achieve an acceptable selection and to elude any failure of the electric appliance. However, it is also suggested to develop a preservation schedule to preserve insulating material in fine status and to prevent any contact with humidity, oxygen and impurities, since they aid in the oxidation process and may result in the reduction of its insulating characteristics and lifespan of the insulating materials and hence the appliance.

**Figure 3:**
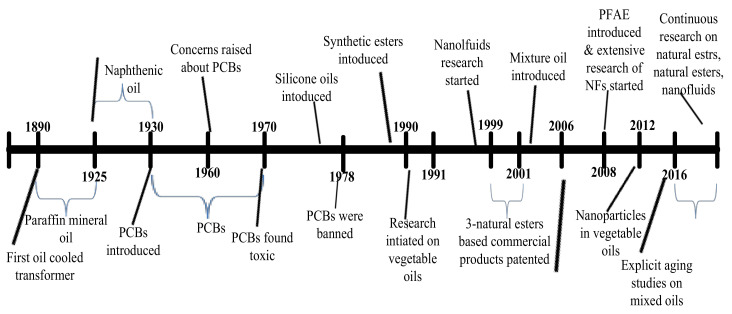
Timeline of research and development on insulating liquids for HV applications.

The major insulating media for HV equipment generally comprises of greatly refined oil and solid insulation. The oil used is essential to be resilient at an elevated temperature that is vital to subdue arcing, to function as a cooling medium and electric insulation [30]. The oil behaves as electric insulation between conductive elements and eradicates heat generated while working on HV equipment. Furthermore, weight, dimension, size and current density of HV equipment relies on the volume of oil and degree of heat transfer [31]. Recently, a wide range of insulating fluids is available for applications in electrical equipment. The lifespan and consistency of HV equipment largely depend on the appropriate insulation structure [32]. The insulation conduct and physical extent of the apparatus are openly associated with the security and consistency of the electric grid. HV equipment is typically consistent during its design lifespan. However, their lifespan may be improved with proper maintenance practices [33]. In the course of working, the insulating system continuously is prone to high functioning temperature and electrical stress. The long term operation of HV equipment at high temperature and high electric field will source obvious effects to the insulation system and hence will lead to operational problems in the normal operation of HV equipment [34]. The degradation degree of insulation arrangement may depreciate with the passage of period within the HV apparatus and this deterioration rate is influenced by electric stresses, temperature, moisture and air [35]. The timeline of the research and development of various types of insulating fluids for HV equipment is given in Figure 3.

The following sections provide a comprehensive analysis of renewable, sustainable and environmentally friendly insulating fluids. It also describes experimental studies on several oil types, including main characteristics, general properties, advantages and disadvantages, applications in HV equipment and associated difficulties and challenges associated with their practical applications.

## 3. Historical Journey Insulating Fluids

The main purpose of an insulating fluid is to ensure insulation and cooling in HV equipment. It must, therefore, possess high insulating resilience, thermal conductivity, chemical stability and should retain the qualities under operation at elevated temperatures and high electric stresses for continued periods [29]. Petroleum-based liquid insulation was first dielectric fluid deliberated for application in HV equipment; though, it was not the first choice as a cooling medium. Based on the literature, an initial oil-immersed transformer was developed in 1890 [36]. The initial crude oil derived liquid was based on small viscosity paraffin oil which delivered outstanding insulation performance; conversely, it demonstrated a high pour point that hinders its applications in HV equipment at lower temperatures. Furthermore, insoluble sludge produced as a result of oxidation would decrease its heat transfer abilities and lifetime [25]. Consequently, paraffin oils were substituted with naphthenic-based oils that indicated low pour point temperatures and possessed greater oxidation stability. The major demerit connected with petroleum-based oils was their high flammability. The accidental leakage may easily catch fire. Fire codes generally demand that HV equipment used inside buildings must be filled with less flammable fluid. These oils are also environmental contaminant, and their dielectric characteristics are promptly degraded by even trivial extent of moisture.

Therefore, researchers made their efforts to develop nonflammable fluid types for explicit applications and presented non-inflammable fluid in the 1930s. An instance of the liquids was askarel or PCB (polychlorinated biphenyls), a collection of synthetic fire-resistant liquids that comprises in their chemical configuration halogenated hydrocarbons with chlorine or fluorine, mainly biphenyls. These were presented as an ideal electric liquid insulation in HV equipment for applications at sensitive places such as shopping malls, hospitals and close to waterways, etc. PCBs have high dielectric strength and not flammable. The askarel was the first time used in the transformer in 1932 and its use continued for approximately 40 years until it was found they were no more ecologically suitable because of toxicity and supplementary ignition derivatives. PCBs were not only toxic but also not biodegradable and very challenging to dispose of securely. When ignited they even develop more toxic and hazardous derivatives. The applications of PCBs were forbidden in several countries at the start of the 1970s, due to apprehensions about the accumulation of PCBs and the harmfulness of their offshoots.

Most equipment using PCBs has been either filled with more appropriate liquids or disposed of under standard recommendations. Thus, HV equipment industries replaced PCBs with MO and high fire point fluids (HFP), for instance, high molecular weight hydrocarbons (HMWH) synthetic esters and silicone liquids [37]. HMWH oils were first HFP substitute liquids which were used to exchange PCBs in HV equipment. HMWH liquids are chemically identical to traditional MO but possess high molecular weight elements in their configuration that brand them tolerant to fire-susceptible spaces and uses. Silicone liquid is polymer-based on silicon whose arrangement consists of carbon, oxygen and hydrogen atoms. It indicates electrical characteristics analogous to those of MO [38], remarkable antioxidative features, exceptional thermal stability and less flammable. Silicone oils were presented as insulation liquid in the middle of the 1970s. They were costly and less biodegradable. Alternatively, synthetic-ester-based liquid insulation were surfaced in 1977 as a substitute for electrical apparatus [39]. Synthetic ester liquids indicated adequate dielectric traits, superior chemical stability and lower pour point. They manifested outstanding properties as compared with MO, for instance, higher fire and flashpoint temperatures and superior biodegradability conduct.

**Figure 4:**
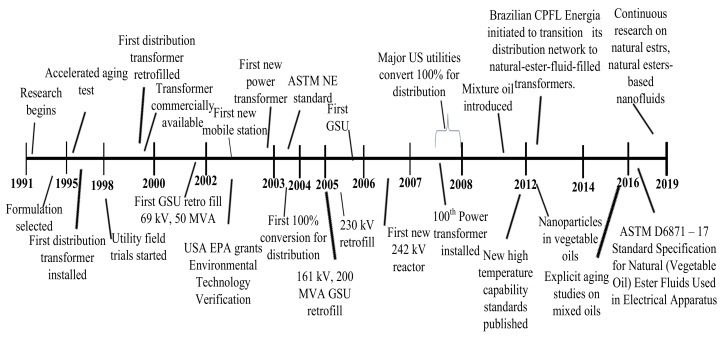
Timeline of proceedings in the development and application of natural-ester insulating fluids.

Lastly, most contemporary development of insulating liquids for HV applications is the renewable, sustainable and environmentally friendly natural esters and pentaerythritol tetra fatty acid natural which usually evolved as a gradually general MO substitute in HV applications mainly in high-fire-risk applications such as indoors or offshore, because of their small volatility and extraordinary fire point [40]. These also have a smaller pour point, high humidity tolerant and enhanced operation at elevated temperature, and these are toxic-free and freely decomposable. VOs also termed as NEs or bio-based liquids are commonly extracted from contemporary entities and derived from plants yields usually sunflower, rapeseed and soybean, etc. [41]. Natural esters developed in the early 1990s in the USA as a “green” and environmentally friendly alternative due to enhanced environmental issues associated with traditional MO and silicon liquids. The first transformer prototype with VOs as an insulating medium was developed in 1996, however standard fabrication of transformer immersed with VOs initiated in 1999 [42]. Timeline events in the development and applications of NEs insulating liquids are given in Figure 4.

Industries and academia are investigating other vegetable-based preparations for liquid insulation such as coconut oils, but still, they are incompatible for applications in cold environments or for high voltages [43]. Investigators are also evaluating nanofluids (NFs) for application as insulation fluid; they would be applied as additives to enhance the dielectric, thermal and chemical stability of insulation.

## 4. Major Features of Insulating Fluids

Dielectric liquids require fulfilling the essential electrical, thermal, physical and chemical traits to ensure satisfactory operation for HV equipment. Usually, the insulating liquid should possess extraordinary dielectric and thermal features, small viscosity, thermal and chemical stability, small ignitability, chemical congruity with other associated substances, mixability with other fluids and ecological compatibility [44]. However, insulating liquids must satisfy specific universal standards to be applied as insulating channels in the HV apparatus, for instance, HV transformers. Common acceptance values of characteristics and usual conduct primarily rely on nature and application of fluid, for instance, in distribution transformers and in the HV apparatus. The properties of liquid insulation to be considered in the development and application in HV equipment are given in Table 1.

Natural esters are obtained from different plant-based seed oils. Their physical and chemical traits rely on their sources of origin, chemical configuration and saturation/unsaturation proportion of fatty acids. The contaminants (moisture, particles, gases) existing in the oil highly impact dielectric strength, which results in the trend to compare them with traditional MOs by observing the contamination level. Water absorption of natural esters and MOs makes a huge difference among them in terms of humidity-absorbing ability; NEs possess more excellent moisture-absorbing capability (20–30 times more) in comparison to MOs which affects the impact of moisture on BDV.

On the subject of security, environmental concerns and thermal characteristics, natural esters ensure better features in comparison to MO. They are categorized as extraordinary fire point liquids with self-quenching traits and appropriate for several industrialized applications. Thermal features and relationship with cellulose insulation contribute toward extended insulation lifetime and permit greater or prolonged overloads, over a longer period without unusual failure of dielectric structures. Natural esters are deliberated as the suitable substitute of MOs in the applications where fire protection, ecological vulnerabilities or better insulation qualities are necessary. Fire safety is one of the main concerns of today’s research community due to the applications of insulating fluids in some sensitive areas for instance, subway channels, ships, offices, shops, workplaces, etc. There are multiple examples of equipment explosion leading to exterior fires that are very hard to quench and may cause the extension to atmospheres due to the seepage of oil. Typical characteristics of natural ester and MO are given in Table 2.

### 4.1. Electrical Properties

Insulating characteristics of insulating fluid differ depending on several dynamics for example moisture content, temperature, applied voltage, frequency level, polarity, electrode configuration, particulates, etc. The application of NEs as an alternative to MOs has multiple benefits. The dielectric constant of natural ester influences the dielectric constant of paper insulation impregnated with ester liquids that are superior to that of MOs. Because of the closer permittivity values of natural esters and impregnated paper insulation, a greater stress is undergone on paper insulation in the case of natural esters liquids than MOs [45]. To choose appropriate insulating fluids, it is essential to identify the dielectric characteristics: breakdown strength (BDV), dielectric constant, dielectric dissipation factor, etc. The dielectric features of various sorts of insulating fluids are presented in Table 3.

#### 4.1.1. Dielectric Breakdown Voltages (BDVs)

The dielectric strength (DS) of insulating material is a measure of its capability to endure electrical stress without collapse. The BDVs of an insulating structure (liquid and solid insulation) is one of the key factors for the electrical structure of HV equipment. It may be defined as the lowest voltages at which there occurs electric conduction that leads to a dielectric rupture. BDV is also termed dielectric strength or striking voltage. The insulation system provides insulation up to a specific voltage extent beyond which insulation BD happens. The voltage at which BD happens is called BDV or DS of insulation. This BDV signifies the electrical insulation competency of the insulation system; thus, a small value manifests that the insulation system is not a good insulation system. The DBV is influenced by the physicochemical properties of materials used, and it is sensitive to humidity and particulate content. Unlike conventional insulation systems (MOs), in which BDV considerably affected by humidity level, environmentally friendly insulation (synthetic and natural esters) retain its dielectric capability at higher humidity levels [47].

All the insulation systems for HV equipment must fulfill AC withstand voltage, lightning impulse (LI) and switching impulse test standards. Numerous investigations have been conducted by multiple researchers to observe the BDV, discharge properties of various insulation systems.

##### AC-Breakdown Strength (AC BDS)

It is an essential condition for the application of the insulation system in HV equipment. The AC BDV is specified as the value of an applied AC voltage at which disruption gets initiated. The oil sample is generally positioned in a test cell/cup and AC voltages are applied across it. The electrodes are sphere heads of standard diameter and separated 2.5 mm apart. The voltages are elevated at a continuously until an arc appeared through the oil between the two electrodes. The voltage at which this arc appears is generally deliberated as AC BDS of the oil. The AC BDV is the uppermost limit of voltage stress that insulation material may survive without failing. AC BDS is highly affected by contaminations present in the insulating fluid, for example, moisture, small particulates, dirt and air or gas fizz. Consequently, measured AC BDV of insulation generally specifies its nature (quality) instead of its features and largely related with fluid composition, it develops superior characteristics when the contamination level is firmly organized. A variety of standard experiment procedures are generally applied where a minute quantity of fluid is exposed to an early consistent electric field between two electrodes dipped into insulation fluid. The voltages are then elevated until BD takes place. AC BDV assessments of insulation fluid must be directed according with standards [48,49]. The main objective of filling HV equipment with insulating fluid is to offer electrical insulation; insulation capacity is governed by composite oil/cellulose insulation scheme. Insulating fluid generally infiltrates into solid insulation and consequently removes air (a smaller BDS than oil). BDS is one of the utmost significant characteristics applied to evaluate the efficiency of insulating liquids. The comparison of AC BDV of different insulating liquids is specified in Table 4.

**Figure 5:**
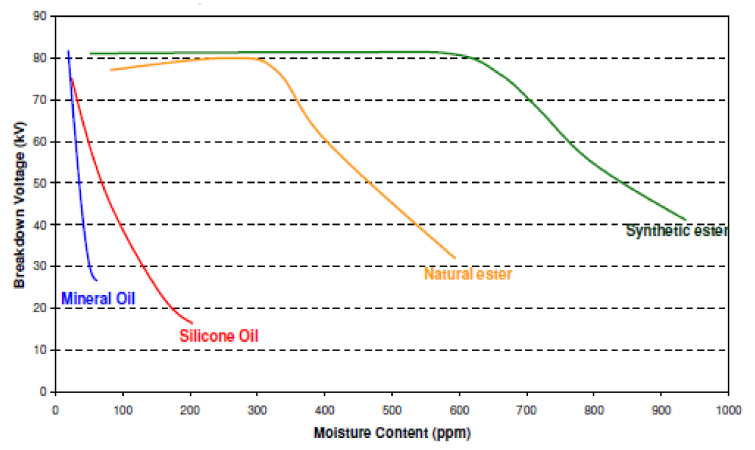
Impact of absolute humidity content on AC BDS of different fluids [5] with permission from Elsevier.

AC BDVs are considerably influenced by the humidity level in the insulating fluid. It may be available in two forms: as free moisture or disintegrated water. Polar liquids have a tendency to develop hydrogen bonds with moisture molecules so that water may simply dissolve. Consequently, polar fluids possess exceedingly high humidity endurance. On the contrary, nonpolar (MO) and marginally polar (silicone oil) is greatly susceptible to the absolute humidity level. The smaller volume of humidity in MO may lead to an expedited decline in BDVs however, ester oils showed high BDS at higher humidity level [51] as shown in Figure 5.

The presence of moisture is hazardous to eh insulation system and hence the overall life of HV equipment. This water may exist in numerous shapes. The existence of moisture in the shape of distinctive drops or as a cloud isolated through fluid may be observed by visual inspection. This form of moisture presence leads to a reduced BDS of the insulation. Darwin et al. investigated the appropriateness of NEs as an insulating liquid for HV equipment and compared their characteristics with MO and SEs [52]. In earlier research, it was found that there is a huge deviation in moisture-soluble degree between VOs and MOs. Better water solubility decreases the influence moisture on the strength of insulation; however also retains cellulose insulation dry resulting in the improved shelf life of a transformer. Contrary, natural esters induce moisture rapidly, and additional attention is required during the use of transformers because of its larger water solubility [52]. Natural esters require distinct operating settings including cleaner and hermetic equipment. In these conditions, natural esters indicated similar or superior insulating performance than MOs [53]. In a current study, Martin et al. concluded that BDS of paper impregnated in natural ester is almost matched with that of paper impregnated with MO [54].

It is noteworthy that the moisture saturation threshold for MOs is not openly pertinent to NE fluids as they are hygroscopic in characteristics and may manage extra moisture and still may preserve BDS to an acceptable value. Nevertheless, the influence of moisture on BDS as a function of percent of saturation is similar for both MO and NE [55].

##### Impulse BDV Test

The impulse strength of the insulation system specifies its capability to endure HV transients of a very short period, for instance, those it may be exposed to through lightning strikes. The standard lightning impulse (LI) specifies simulating lightning shots and usually uses 1.2-μs surge for a wave to achieve a 90% amplitude and decline down to 50% amplitude after 50 μs. The LI BDV is generally examined by IEC 60897 standard.

Contrary to AC BDV tests, impulse BD test is not largely influenced by water content and particulate impurities in the liquid insulation consequently may be applied to evaluate the dielectric features of the insulating liquid itself. Generally, two standards (ASTM D 3300 and IEC 60897) are mostly used for impulse BDVs of insulating fluids. In a study [56], the impulse BDV measurements for various types of fluids insulation have been conducted with various gap distances and the results of this investigation are given in Table 5.

##### Impulse BDV of Alternative Liquid Insulation Impregnated Cellulose

Impulse BD experiments on Kraft paper impregnated by MO and ester dielectric liquids were investigated by applying plate–plate electrodes, in accordance with ASTM D3300 test standard [51]. These findings are in consistent with impulse BDVs of alternative liquid insulations, indicating superior BDS than that of MO.

#### 4.1.2. Partial Discharge (PD) Tests

For a nonuniform field with relatively bigger oil gaps, PD tests are normally applied rather than AC BD tests. PD is a localized electric discharge that does not bridge the insulation between two conducting electrodes. The voltage level when ionization and PD begin to occur is known as Partial discharge inception voltage (PDIV). PD activity may happen at any point in the insulation system, where electric field strength surpasses the BDS of that part of insulating material. PD also performs an important role in the acceleration of thermal aging and of deterioration of dielectric liquid. PD performance has been investigated for the insulation system by multiple researchers and still research is going on to is still in progress to study the PD conduct of these fluids. Mohamed et al. investigated the PDIV of MO and NE by using a needle to plane electrode configuration. They concluded that NE oil has superior PDIV than MO and indicated great potential for the MV transformer insulation system [57].

#### 4.1.3. Dissipation Factor (DF)

The DF is the amount of dielectric loss happening in dielectric fluid when it is exposed to an AC field. The DF usually surges with an increasing existence of impurities or aging derivatives for example, humidity, carbon or additional conducting substances and oxidation derivatives. Power factor (PF) designates the dielectric loss in the insulating liquid and therefore the extent of energy dissipated as heat. This PF test is largely applied as a preemptive preservation experiment for approval of dielectric liquid. In the end, it is the incompetence of dielectric liquid molecules to reorient when they are exposed to an alternating electric field, turning into the form of heat losses. PF is influenced by contaminating agents for instance, moisture, solvents and conductive particulates [58,59]. Consequently, an insulating liquid with a large value of PF manifests that it is depreciated or polluted. Ecologically friendly liquids such as natural esters indicated high PF than nonpolar MOs, particularly at higher temperatures [60,61,62].

#### 4.1.4. Permittivity

Dielectric constant or permittivity is associated with the capacity of the insulating liquid to transfer an electric field. It may be deliberated as a feature prone to polar impurities; therefore, small values of permittivity may designate the existence of impurities, for example moisture or particulates and modifications in oil composition, e.g., oxidation, deterioration or additive consumption. The electric voltages in a complex scheme are divided between the liquid and cellulose insulation in terms of permittivity of materials. In the case of an insulation system with traditional MO and solid insulating material, in which dielectric constant of solid is larger than that of liquid, an electric voltage is larger in entire oil is greater than through solid material. Ecologically friendly liquids referring to esters (synthetic and natural) possess dielectric constant generally larger than cellulose insulation (paper and pressboard). As a result, in the transformer insulation structure with ester liquid, the discrepancy in the sharing of stresses among solid and fluid is considerably smaller than MO insulating system [63].

### 4.2. Physical Properties

Every insulating fluid has extensively different physical traits—particularly viscosity, density, pour point, interfacial tension and flashpoint, as indicated in Table 6.

#### 4.2.1. Color and Appearance

The color and appearance of freshly inserted insulation fluid are light and clear. With the passage of time and functioning, the color of liquid insulation transforms into dark color and in multiple circumstances, this is the primary sign of degradation or unhealthiness of oil.

#### 4.2.2. Relative Density (Specific Gravity)

Density is an important physical feature that may be used in conjunction with other properties in various chemical industrial applications. The specific gravity of liquid insulation is specified as the ratio of the weight of oil and water of equivalent quantities. The relative density of a fluid is the ratio of weights of equivalent amounts of fluid and water at 15 °C. It is significant to measure the specific gravity of liquid insulation in specific uses, for instance in a freezing environment, where ice may develop within the transformer container if it is exposed to temperatures under the freezing. In these circumstances, ice float along topside oil could lead to a flashover between conductors over oil level. The density of natural esters shall be determined according to ISO 3675 (reference—international procedure). Table 7 indicates the relative density bounds specified in various standards for MO and NE fluids.

Physical characteristics such as viscosity and density perform a vital role for the investigation of insulating fluids. However, there is little research available indicating that optimization of temperature in natural esters can enhance their performance in electrical equipment. Therefore, it may be useful to identify the significance of these properties within a broad temperature range of every natural ester. Forma et al. [28] investigated the density of different natural ester types at several temperatures, as indicated in Table 8 [46,67,68].

#### 4.2.3. Viscosity

The viscosity of a fluid is specified as an amount of opposition to its flow. Kinematic viscosity (KV) is the most important factor for heat transfer. The viscosity of oil influences the ability to transfer the heat by conduction; therefore, cooling the electric equipment by conduction is major heat removing framework. In dielectric liquids for HV applications, this feature is narrowly associated with heat transport efficiency; thus, an insulating liquid with small viscosity is extremely necessary as a means to attain effective cooling performance in HV equipment [69]. The smaller value permits a higher rate of heat transfer in the HV equipment such as the transformer. Viscosity is computed by determining time engaged by a specific quantity of oil to pour within a calibrated tube. The heat transmission capability of fluid is hugely affected by its viscosity. Fluid with high viscosity has considerably decreased cooling performance. Low viscous oil enhances cooling efficiency by easy circulation. More important, in a cold environment, oil with high viscosity will obstruct heat transference from hotspots because of its poor distribution capability and also influences the velocity of moving components (such as in circuit breakers and tap changers). The viscosity of liquid insulation likewise influences fluid treatment and solid impregnation period.

The higher viscosity of liquids gives rise to greater hot spot temperatures inside electrical equipment. Rycroft [30] stated that the application of VOs in the transformer results in a rise in temperature by 1–3 °C. The viscosity of dielectric fluid influences heat transferal capability by conduction. Conduction cooling is primary heat elimination tools in HV equipment especially transformers and high viscosity may give rise to greater hot spot temperatures inside the equipment.

Environmentally friendly ester-based liquids are classified to demonstrate superior viscosity values in comparison to MOs, being this variance more significant at a lower temperature. The larger viscosity of NE liquids would affect the thermal configuration of transformers, as the flow of liquid within the core, winding and cooling tools is lesser than MO, resulting in a relative rise in top oil, windings and core temperatures [70,71]. In contrast, a vital feature of NEs is that oxidation development initiated extraordinary concentrations of oxygen and high operation temperatures enhance its viscosity that reduces its heat transferal ability, leading to insulation overheating [72]. Experimental investigations have specified that the use of VOs in transformers caused in elevated temperatures between 1–3 °C [73].

In general, at the normal working temperature of electric equipment, natural esters indicate larger viscosity than MOs and lower than silicone oils. The research work shows that heat transfer in equipment by convection is lesser effective with natural esters. For an efficient cooling system for electric equipment, cooling system design and viscosity must be controlled. Higher viscosity results in a deduction that the cooling efficiency of NEs is lower to MO, however it is not essentially correct. Due to higher specific heat ability and heat thermal conductivity, VOs are capable of transfer a greater extent of heat. Consequently, cooling conduct over the lifespan of electric equipment may be a benefit if a cooling scheme is adapted to the traits of NE.

The viscosity intensely affects the permeation of solid insulation. Normally, the viscosity of NEs is fourfold higher than MOs in the temperature range (almost 70 °C). Thus, the mechanical scheme of huge insulation configurations along with the transformer impregnates the route required to adapt as per demand. For instance, the structure of huge woody and pressboard components must be reexamined during applying esters as an insulating agent. The establishments of drying and impregnation openings are recommended to expedite impregnation action. Impregnation experiments of pressboard suggest that it is required to prolong the impregnating period if the identical boundary settings (e.g., fluid temperature) are retained. Because of olefin of fatty esters and infrequent contact to atmosphere and heat, the viscosity of natural esters may enhance oil aging [74]. It was witnessed that with the application of NEs, it requires as a minimum two times greater to accomplish the permeation course of layered pressboard. The fluid temperature for duo samples is identical; NE and synthetic esters act in the same way due to comparable viscosity conduct. With regard to accelerating the course of permeation, ester fluid must be treated at marginally elevated temperatures. Nevertheless, the fluid temperature has to restrict subject to the configuration of natural ester. In particular, for NEs, additions would be expended at a greater temperature in vacuum circumstances. It is suggested to prolong the impregnation period and raise the temperature a little of natural esters to execute an effective transformer application process. This approach will be helpful for the appropriate impregnation of solid insulation material.

#### 4.2.4. Pour Point

Insulating fluid generally circulates within the HV equipment for the intention of customary natural cooling. Therefore, the appropriate flow of this liquid insulation is significant to provide the required cooling of the equipment. The pour point of fluid insulation refers to that lowermost temperature at which insulating fluid merely initiate to flow/pour easily, when examined under prescribed conditions. The pour point of liquid insulation is specified as temperature whereat fluid rejects to pour/flow under specified experiment circumstances. Information about the pour point of the oil is vital to evaluate its appropriateness for application in specific weather. Pour point is a valuable measure to know how insulating fluid will execute at low temperatures specifically while this is crucial to startup a transformer in extremely cold conditions. When the temperature of insulating fluid falls under the pour point, it inhibits convention flowing and hinder the cooling of the transformer. Furthermore, the movement of tap changer may be impacted. Paraffin-based liquid insulation has a high pour point due to higher wax content [75]. Measurement of pour point is significant as it directly related to the cooling of HV equipment. Environmentally friendly liquids possess greater pour point than MO, whereas synthetic esters possess pour point close to that of conventional MO. A simple and cost-effective solution to this problem of natural esters is to incorporate pour point depressants [76]. Manufacturing practice variation is an additional substitute deliberated to enhance cold conduct in natural esters [77]. VOs indicate a larger pour point than MOs usually in scale of −15 to −25 °C however, the trial has shown effective cold launch extending to −30 °C [73].

Insulating liquid applied in electric equipment behave as both coolant insulator for interior elements and is projected to endure adequate flow throughout intense temperature situations (hot or cold). Supplements such as PPDs (Pour Point Depressants) and HMWH (high molecular weight hydrocarbon) are added to natural esters to improve their conduct at low temperatures [78,79]. Acid chain span, unsaturation and sectioning are morphologic characteristics that may be useful to regulate the pour point of natural esters. Together unsaturation and branch off in fatty acid possess an encouraging effect on dropping pour point. Unsaturated fatty acids are additionally efficient in decreasing pour point than branched fatty acids of identical carbon chain length. The existences of aromatic assemblies in NEs are also favorable in maintaining lower pour pint. Table 9 provides pour points limits specified in various standards and also indicates the cold start performance of various insulating fluids.

#### 4.2.5. Flash and Fire Point

The flammability of HV equipment is the most important security worry during recent times. There are numerous instances of HV apparatus outbursts leading to flames that are hard to quench and extend to nearby regions as fluid outflows. Flash and fire points are measures of fluids opposition to ignite a fire. The flash point defines the tendency of fluid insulation to establish a combustible compound with an atmosphere under ordered laboratory environments. This experiment offers one characteristic of evaluating the total flammability vulnerability of a material. Flash point is applied in shipment and security guidelines to describe flammable and combustible materials. Flash point may designate the potential existence of highly impulsive and flammable materials. The most significant benefit of natural esters is their relatively larger fire and flash points than presently accessible MOs. Fire points perform vital part throughout the conveyance of fluids and setting up transformer in indoors or in practical structures with or in the absence of additional ignite safety measures. NEs possess fire points of almost 360 °C and entitled as the “K” category according to IEC 61100. Natural esters are being applied for multiple electric facilities and possess supervisory benefits at several locations [83,84]. Chandrasekar et al. studied flash and fire points of MO and NE (sunflower and rice bran oils) and concluded NEs have superior to MO. Mineral oil and Natural ester (Rice bran oil and Sunflower oil) are analyzed and from the analysis, it is concluded that vegetable oils have better breakdown voltage, flash point, fire point, Density of oil, than the mineral oil.

The fire defiance efficiency of insulating liquid is frequently categorized with regards to flash and fire points. The flash point of insulation fluid is temperature whereat fluid surface is blistered and creates an adequate vapor which would develop a combustible blend with atmosphere. It is temperature whereat oil exterior radiates sufficient fumes to ignite in the existence of a flame. Contrary, the fire point/flame point is temperature whereat fluid surface fumes would flare up and withstand for a minimum of five seconds in the presence of flame. The fire and flash points of liquid insulation are, therefore, valuable evidence in defining impulsiveness and fire resistivity characteristics of the fluid. A small value of flash point would usually specify that fluid has explosive ignitable impurities within it. Table 10 presents a comparison of fire defiance characteristics of MO and NE fluids. NEC^TM^ wants a fire point of as a minimum 300 °C to categorize as “less inflammable fluids”. Conventional MOs possess small flammability, whereas ester-based liquids and silicon are acknowledged as “less-flammable fluid”. Ecologically friendly liquids have considerably larger flash and fire points (˃300 °C) than MO; consequently, these are categorized as K type liquids conferring to standard IEC 61100. Natural esters have manifested considerably larger flash and fire points than traditional MO. The calorific value of dielectric fluid is a degree of extent of energy created by thorough combustion of the material investigated. There would have no stated instances of equipment fires containing NE fluid. The flash and fire points are enormously susceptible to impurities with impulsive composites or contaminants of smaller inflammability; thus, smaller values may be applied to deliver qualitative symptoms of impurities in a dielectric liquid. Relative values for various fluid insulations are presented in Table 11. It is evident that the value of natural ester is more than 300 °C than 170 °C for MO. Relative evaluation of various insulating fluids is given in Table 12.

#### 4.2.6. Interfacial Tension (IFT)

IFT is characteristic of fluid that is a measure of the surface tension maintained against water under non-equilibrium situations. Such a feature indirectly designates the volume of oil-soluble polar impurities and oxidation derivatives existing in the dielectric fluids. The layer resilience of fluid is furthermore deteriorated while specific pollutants for instance, paints, soaps, varnished and oxidation derivatives, exist in the fluid [85].

#### 4.2.7. Operating Temperature

The functioning temperature of HV equipment affects the lifespan of cellulose insulation that deteriorates at proportions relying on both temperature and dielectric fluid. The investigational outcomes showed that with vegetable oils, this is likely to run a transformer at greater temperatures than with MO. The temperature is not evaluated as average, but as “hot spot” temperature in transformer coils. Higher functional temperatures meant augmented overloading of the transformer, a significant concern while regarding at a present power station.

#### 4.2.8. Water Assimilation/Water Saturation

The existence of water in the insulation system is hazardous for the general life of HV equipment. It could be found in insulating fluid in various shapes. The existence of moisture in the shape of isolated drops or as a cloud isolated across the fluid may be observed by a visual investigation. This kind of moisture existence leads to reduced BDS of fluid. This is significant to state that moisture congestion limitation for MOs is not openly valid to NE fluids as they are naturally hygroscopic and could manage additional moisture content and still can preserve DS to an acceptable value. Therefore, the influence of moisture level on DS in terms of percentage of engorgement is similar for both NE and MO [55,86].

The water absorption of insulating fluid is the overall quantity of humidity content that fluid may grasp in the absence of free water being submitted at room temperature. The solvability of freely moisture surges with a rise in temperatures in whole kinds of liquid insulations. NEs oils may enhance thermal permanence of cellulose, as they eradicate additional humidity from paper insulation more efficiently in comparison to MO permitting either higher spot temperature or enhanced apparatus lifetime. The water absorption of various insulating fluids at room temperature is given in Table 13.

### 4.3. Chemical Features

Chemical features of a dielectric fluid are one of the utmost significant factors which impact the operation of HV equipment in the longer term. In recent times, it is identified that an insulating fluid must be able to provide a good balance between high operational performances, fewer environmental problems and within the equipment, it must be chemically and thermally reliable and possess outstanding dielectric characteristics. Chemical features of dielectric fluid for example, moisture level, acidity, oxidation permanence, aging properties and erosive sulfur, are extremely crucial. Table 14 indicates significant chemical characteristics of various insulating fluids and comprehensive analysis is as follow:

#### 4.3.1. Acidity (Neutralization Number)

One of the primary factors for insulating liquid is acid number (neutralization value, whole acidity). Neutralization number is a gauge of acidity that applies to the number of milligrams of potassium hydroxide needed to counteract the H+ ions in one gram of fluid. The pureness of treated fluid may be assessed by specifying the total acid value. The acidity is the number of acidic constituents existing in a dielectric fluid. Afresh and unused liquid insulation is projected to be unbiased and free from any acidic substance. In the course of service, acidity is the portion of acidic derivatives of oxidation of fluid. The acidity and consequently neutralization number rise as oil age through an operation. A high acid number designates the existence of oxidation or impurity with external substances. The neutralization number may be applied as a valuable gauge if fluid must be reused or changed. Supervision of acid value all through the operation is a vital parameter to confirm the safe working and conduct of electric equipment. There are some studies accessible on the acidity of natural esters fluids. Oxidation stability of insulating fluid may be examined by knowing acidity, dielectric dissipation factor and specific resistance. NEs endure oxidation and likewise disintegrate by hydrolysis resulting in the development of derivatives (acids and alcohols). Therefore, finding the acidity appears relevant, recently standard experiment procedure is not accessible for VO—and currently—test technique (IEC 62021–1) for MOs would apply for natural esters. Ashraful et al. concluded that the acidity value of VOs rose consistently with time [87]. Obadiah et al. attained the same results for methyl esters [88].

#### 4.3.2. Oxidation Stability

Oxidation stability of dielectric liquids is a crucial factor since this is highly necessary that liquid should not be oxidizing with the passage of time. The reliability of dielectric liquids is considerably influenced by oxidation and aging mechanism that effect openly in equipment’s lifespan. The oxidation stability of the insulating fluid is a feature that specifies its resistance to oxidation during operation. The oxidation of liquid insulation is a critical matter since it leads to the development of derivatives, e.g., acids and sludge, which in turn originate issues in the apparatus by decreasing dielectric features of solid insulating material [89]. A large value of oxidation stability is desired to certify extended service lifetime of insulating fluid. Else, oxidation of fluid leads to acidity and sludge development, causing amplified electric losses, metal erosion and electric failures, thus decreasing insulation functioning lifetime.

In both NEs and MOs, oxidation development is a serious issue, however due to numerous factors, NEs are inappropriate for applications in free-breathing transformers because of critical variation in viscosity. The natural esters should not be exposed to atmospheric contact and sludge formation in transformers. MOs also suffer from the oxidation process throughout service resulting in the development of sludge, but adding antioxidants may overdue the process.

The existence of Carbon–Carbon dual bond converts dielectric fluids susceptible to oxidation. In NE, additional carbon–carbon dual bonds are existent than MO and it is largely prone to oxidation. The oxidation procedure is irreparable and reaction action, oxygen is expended. The oxidation stability of insulating fluids is a key apprehension of end consumers. As a function of their relative oxidation stability, silicone liquids are acknowledged as highly stable dielectric liquid succeeded by synthetic esters, then mineral oils and finally NEs [90].

Natural esters indicate low oxidation stability than their counterparts (MOs). Nevertheless, the process of oxidation in natural esters varies from MOs. In the case of natural ester fluids, constant contact to oxygen does not develop any solid precipitates. Instead, it will develop complex molecules that may somewhat enhance the viscosity of vegetable oil in the tank and/or generates oxygen comprising derivatives for instance acids, alcohols and ketones and ultimately leads to polymerization. With constant contact with oxygen, a fine layer of gel is developed in NE.

The characteristics of NEs may be stabilized by selecting a suitable configuration of saturated and unsaturated triglycerides (TGs) during preparation. Nevertheless, dual bonds have been extra sensitive to oxidation; unsaturated TGs are lower stabilized for oxidation than saturated ones; however, saturated TGs indicate large pour point and viscosity. Altogether these deliberations are vital to choosing better natural esters for HV applications.

To evade the oxidation of natural ester fluid throughout production route along with functioning, it is required to hinder contact of dielectric fluid to air. As a result of this ester fluid filled transformers are manufactured with sealed type construction [66]. To confirm and sustain optimal conduct of VOs, contact to humidity and oxygen should be curtailed. Therefore, airtight closing against ambient atmosphere is the finest solution to take advantage of the aforementioned features of VOs [59]. Antioxidants additives are often used by suppliers as inhibitors that decelerate oxidation in insulating fluids, thus enhancing service life expectancy. Huge transformer’s designs frequently use nitrogen in headspace or those with dielectric fluid conservators need a membrane wall between dielectric fluid and outer venting. The use of natural esters is recommended in free-breathing units owing to this oxidation issue [54].

Oxidation stability of natural esters should be enhanced by the application of antioxidant additions. The oxidation progression takes place via free radical technique wherever antioxidants behave as radical scavengers disintegrating the propagation phase of course. Oxidation stability of VOs relies on the dissemination of fatty acids, filtering action, existing organic/natural antioxidants. This is likewise known that the oxidation stability of natural esters reduces with a rise in unsaturated fatty acid level. Oxidation stability of oleic acid (one double bond) is stated tenfold larger than linoleic acid (two double bonds) whereas linoleic acid is towing times more stable than linolenic acid (three double bonds). Oxidation stability of VOs is also influenced by the cleansing progression that constitutes multiple phases such as decreasing the volume of free fatty acids, waxes, metals, coloring dyes and aromas and also participates in reducing organic antioxidants volume. Table 15 gives the stability particulars specified in different studies for insulating fluids.

#### 4.3.3. Aging Characteristics

Aging of insulating fluids results in degradation of insulating properties depends on time, temperature, humidity and oxygen amount. Due to contemporary hermetic structures, the impact of water and oxygen contribution has decreased, although the temperature is still the major controlling factor for accelerated aging. Oil-immersed transformers mainly include insulating fluid, cellulose insulation, iron core and a copper coil. Each of these elements influences the deterioration process of insulating liquid.

Mc Shane et al. conducted the accelerated aging investigations and concluded that less decomposition in transformers loaded with NEs in comparison to transformers loaded with MOs [91]. In another inquiry, Choi et al. found that the total acid number in aged natural ester oil was greater than MO. Nevertheless, acids developed appear to be not detrimental to cellulose or fluid insulation. The aged VOs include long-chain fatty acids, and the existence of these acids in NEs is non-erosive as compare to short-chain organic acids existed in traditional MO. Thus, the neutralization value in NEs does not lead to any variation in insulating performance [92].

#### 4.3.4. Furans Content

Furanic substances are deterioration derivatives of solid insulating constituents (insulating paper and pressboard). These materials frequently resolvable in insulating fluid and therefore, an estimate of furanic substances in fluid shows the degree of cellulose decay. Liquid chromatography (HPLC) is used to measure furans content in fluid insulation. New insulating fluid must not possess any noteworthy amount of furanic materials disintegrated in it [93].

#### 4.3.5. Corrosive Sulfur Content

The existence of sulfur or sulfur comprising combinations in the insulating fluid may result in erosion of polished copper coils. This may whatsoever originate from crude or from the rubber hoses that are used in the course of oil processing and even it may emerge from gasket materials. It is usually recognized that the existence of corrosive sulfur substances and issues they originate in electric equipment (especially transformers) are intrinsically a severe concern in MO. Natural VOs are extracted from seeds of harvests and also do not comprise sulfur that leads to the corrosiveness of metallic constituents in the HV apparatus. Therefore, these insulating liquids would not create erosive complications in the transformer or other HV equipment [25]. Sulfur may be initiated into the transformer through causal ways, for instance, via application of mismatched hosepipes [26]. Rapp et al. [27] studied characteristics of transformer occupied with erosive sulfur contaminated oil and sulfur-free natural ester liquids. Suspension of passivation to retro- loading of natural ester decreases the impact on corrosive sulfur. It was established from an analysis that natural ester liquid doped with passivators reserved the features as per ASTM D6871 limits.

#### 4.3.6. Total Gas Content/Dissolved Gas Analysis (DGA) and Stray Gassing

Under electric and thermal stresses, insulating fluid and fluid-impregnated insulating materials may deteriorate and produce gaseous disintegration derivatives that disband into the fluid. The quantity, structure and degree of production of these disbanded gases assist as a worthy index of category and sternness of defects or irregularities occurring within the equipment.

Stray gassing is specified as” production of gases when dielectric fluids are heated at reasonably low temperatures (90 °C–200 °C) [94]. Table 16 provides the modification in DGA techniques and Duval Triangle for their application to evaluating NE and MO under diverse fault situations. In NE at comparatively low temperatures (80 °C–250 °C), a significant formation of stray gases such as hydrogen and ethane are witnessed for a specific time period (weeks to months) subsequently, the transformer is activated [82]. NEs manifest larger gas development on account of PD (hydrogen with hints of acetylene) as compared with MO at similar voltage value [95]. 

### 4.4. Environmental Properties

The most significant concerns with insulating liquids may include their renewability, biodegradability and their respect for environmental guidelines in the event of leakage and accidental fire. MOs are not biodegradable and possess adverse environmental profile, which specifies they are harmful to humanoid and marine life (in case of spillages). Therefore, substitute liquids must obey environmental protocols and would not be perilous. Multiple investigators have examined environmental and fire-related effectiveness of natural esters liquids. The following section presents inquiries of these parameters in ester dielectric liquids with MOs. The environmental traits contain key factors for instance, biodegradability, toxicity and sustainability, whereas fire characteristics may constitute flash point, fire point and emission outline of dielectric liquid.

#### 4.4.1. Biodegradability and Toxicity

Ecological protection of a dielectric liquid is generally evaluated by two aspects: biodegradability and low toxicity. During the preceding few years, the criterion has attracted huge consideration because of spillage and leakage calamities of dielectric liquids of transformers and associated adverse effects on water and soil. Commonly, liquids that have a prompt biodecomposition degree and low toxicity are categorized ecologically friendly and are vital at the instant of in view of the application in environmentally delicate sites. In general, during the transformer collapse, the dielectric liquid seeped to soil may be the origin of its impurity and as a result, there is also contagion of waterways flora and fauna. Biodegradability is a gauge of the perseverance of any material or component in environs and is a base point for evaluation of the ecologically friendly matters [97]. Normally, fluids which possess larger biodegradation percentage and exhibit smaller toxicity are categorized as “environmentally friendly”. These dynamics are significant while looking to apply insulation materials in ecologically sensitive regions, for example in water passages, buildings, shopping malls to evade defilement. The word “biodegradability” reveals the degree by which oil is digested by happening organic microorganisms in soil or water paths, in the occasion of leakage or seepage. Obviously, it is worthy if seeped fluids fade naturally without the application of costly cleanup actions. Conventional MOs and silicone oils have very poor environmental conduct; their degradation is not higher than 30% and 10%, respectively, accordingly, such liquids are not termed as biodegradable liquids. Alternatively, natural esters demonstrate an exceptional performance (˃95% of biodegradation); hence these are categorized as possessing freely and definitive biodegradability as these fulfill definite experiment conditions of rapid biodegradation [98]. Synthetic ester also has high environmental conduct, however, lesser than natural esters as they approach 70%–80% of biodegradation; so, these are categorized as freely biodegradable [8]. Ecologically friendly esters are similarly acknowledged as nonhazardous and harmless to marine lives [50].

The environmental properties of natural ester are outstanding as they undertake biodegradation very swiftly and entirely and generate nontoxic derivatives. Natural esters don’t possess semi-unstable organics, hazardous aromatics, volatiles, halogens, etc. The huge biodegradability rate of NEs facilitates a monetary advantage for other capacity ventures because of no isolated containment capacity required to be set up to avoid environmental pollution in the occurrence of seepage.

Biodegradation is a process in which natural materials deteriorate to smaller molecular weight matters by enzymes generated by the course of microorganisms. Organic substances experience deterioration in the existence of oxygen (aerobic) and in deficiency of oxygen (anaerobic). The procedure of transformation of an organic substance into inanimates is called as bio-mineralization [99]. There are generally two kinds of biodegradation. First is named main biodegradation, recognized as a conversion of a matter by act of microorganisms such that modification is introduced in certain definite assessable characteristics of the material that modifies natural traits of a substance, although parting molecule mainly unharmed [100]. Intermediate metabolites formed may, though, be lethal than the original substrate [45]. Second is final or thorough biodegradation accomplished when a material is entirely consumed by microorganisms leading to the development of low molecule weight derivatives such as CO_2_, methane, H_2_O, minerals and the latest bacterial cellular elements that are known as mineralization [97].

There exist several trial systems for biodegradation investigations of substance that evaluate loss of disbanded organic carbon materials and endorsement of oxygen because of the act of microorganisms and quantity of CO_2_ generated during a particular time and there are, however, other methods, the application of oxygen through microorganism activity [101]. 

#### 4.4.2. Emission Profile

The emission profile is a significant indicator of the environmental behavior of any dielectric liquid [102]. It designates character and varieties of fumes discharged and also conducts of fire while the liquid is exposed to ignition. Fire and flashpoints represent the highest temperature whereat oil can be securely applied without the considerable hazard of fire. Ideally, liquids must merely burn at extraordinary temperatures and the fumes released must not harm health and the environment. MOs are mixtures of hydrocarbons and liberate lethal gases when burned. On the other hand, nature esters are groupings of acids and alcohols extracted from natural methods and are ecologically friendly [103]. Table 17 shows the environmental characteristics of various insulating fluids.

## 5. Performance, Evaluation and Analysis of Renewable Oils as Insulating Fluids

MOs generally used as dielectric fluids in transformers, are acquired by crude oil refinement and subsequent processing. The concluding properties of traditional MO are determined by chemical structure [104]. In contrast, vegetable oils are triacylglycerides which are associated with a panel of organic blends that are formed by the reaction of an acid with alcohol [105]. The viscosity of VOs is comparatively greater than MO and it is fairly offsetting by supplementary thermal features; for instance, thermal scheme of transformer is analogous for both MO and VOs. Variation in chemical configuration demonstrates differences in liquid features, viz., viscosity, thermal conductivity and heat capability of liquid may improve cooling scheme of a transformer. The use of natural esters is growing due to their benefits over MOs. Natural esters have extraordinary biodegradability; consequently, they are categorized as environmentally friendly [106].

### 5.1. Electrical Properties

The BDS of fluid is influenced by moisture quantity existent in it. It is concluded by many researchers that the BDS of NE oils is less influenced by humidity than MO [107,108,109]. Dijin Divakaran et al. investigated the insulating properties of MO (virgin and aged) and natural esters (fresh and aged) and concluded that natural esters presented superior dielectric insulation features [110]. The outcomes of AC voltages test for natural ester-immersed pressboard were superior to the MO-immersed pressboard [111]. Hemmer et al. tested various samples of oils and concluded that rapeseed oil (RAPSOLT) have better congruity with conventional MO; specifically, the outcomes of AC BDS of RAPSOLT-impregnated pressboard were quite superior. Furthermore, accelerated aging of natural esters did not impact dielectric losses as high as for MOs [112]. From the findings of BDS, it may be concluded that natural esters could be a great substitute for insulating fluid in HV equipment especially transformers.

Sitorus et al. investigated streamer propagation occurrence in jatropha curcas methyl ester oil (JMEO) and MO under LI voltages. The negative streamer in MO is greatly filamentary in comparison to JMEO. There was no apparent dissimilarity between positive streamers profiles in both of the tested fluids. The author concluded that JMEO oil could possibly be an alternative for MO for liquid insulation in power transformers [113].

Chandrasekar et al. carried out a relative study of PD properties of thermally aged fluids (vegetable oil and MO). This study composed standard PD diagrams from vegetable oils, thereby observing their applicability in HV transformers [14]. The impact of thermal aging on BDS appears much lower on vegetable oils in comparison to MO. Lesser PD events were observed in palm and corn fluids than MO. NEs have great potential to replace MO from the perspective of PDIV when fresh, refined and greatly filtered liquids applied. The minor permittivity disparity between pressboard and NE, compared to that of between pressboard and MO, result in a more uniform field distribution at oil/pressboard interface. NE indicated higher PDIVs than MO. Nevertheless, knowledge about the oil/pressboard is inadequate. There is a deficiency of standardization in PDIV measurements. Meanwhile, the industry is struggling to offer innovative liquid insulation for application at higher transmission voltages, advance testing practices, and awareness of the oil/pressboard interface is important [114].

### 5.2. Physicochemical Properties

The fluids used in HV equipment for insulation and cooling are subjected to thermal and electric stress. Divakaran et al. examined the thermal properties of various insulating fluids and concluded that natural esters presented superior thermal behavior. Palm and coconut oil indicated safer fir and flash point [110]. Takaaki Kanoh et al. conducted stability tests and indicated that there was small variation in both total acid value and BDV of natural ester (PFAE). As a result, the oxidation stability of PFAE is found better than MO [115].

Natural esters have some serious issues with their oxidation stability; therefore, hermetically sealing could be a suitable way to endorse the steady function of the transformer using eco-friendly liquids. The lifespan of the transformer and its insulation system may be prolonged by a blend of two techniques viz., preventing the exposure of oil with moisture or using a vegetable oil with the greater capability of moisture saturation. The fluids may encounter thermal and electrical stress; accordingly, this is vital to identify the impact of these pressures. Gas production is a convenient measured feature, and this is significant to determine the production of gas post aging in the incidence of Cu for the definite time duration. The key difference in disintegration yields, than hydrocarbon fluids, is the creation of CO and CO_2_ in enormous extent. This is mainly due to carbonyl set -COO that split down to provide CO and CO_2_. This was determined that whole gas generated by natural esters was one-quarter of gas created by MO. This manifests the arc-quenching capacity of natural esters is high [110,116].

Vegetable oils disintegrate moisture almost twentyfold higher than a MO at any specified temperature [109]. Limits for water in MO must thus not be directly used for natural esters. For example, the impact of water on dropping the BDV of fluid is more associated with the percentage of saturation instead of water volume [80]. The moisture level of fluid is generally demonstrated as either mg/kg (mass water/mass oil) or as moisture activity (vapor pressure of soluble moisture/vapor pressure of pure water at a specific temperature). The moisture content of natural esters considerably differed from MO. At ambient temperature, moisture congestion of MO is almost 60 mg/kg while NEs are almost 1000 mg/kg [87] BDV of a fluid initiates to decline while comparative saturation increases to almost 40%–50%. Rather than applying a percent saturation, absolute water content permits a guide correlation between NEs and MO. Electric and physiochemical characteristics of NEs particularly designed for the transformer is given at Table 18.

## 6. Technical Complications and Future Research Agenda

### 6.1. Technical Complications

Significant practical complications that surfaced during the practical applications of natural esters in HV equipment are summarized as follows:

i. The price of natural esters oil is generally dependent on the feedstock cost;
ii. Storage and management of natural esters are problematic and very challenging;
iii. Homogeneity of products is generally determined by the trader, starting material and fabrication procedures;
iv. Approval by equipment industrialists is an additional problem;
v. Uninterrupted accessibility of natural esters is required to be ensured launching on the main consumption of it in HV equipment;
vi. Natural esters application in HV equipment subjected to freezing environments, has been a serious concern. The pour point of natural esters in no way proceeds beyond −30 °C even following the addition of depressants. Excluding additions, oil may frost at below zero temperatures;
vii. HV equipment (especially transformers) filled with natural ester oil must be airtight to inhibit the entrance of humidity and air into the unit. Antioxidants must be present in the sealed unit due to the potential inclusion of the mentioned impurities throughout the lifespan of the unit.

### 6.2. Research gap and Future Research Agenda

The large proportion of research on ester liquids has emphasized on studying their deterioration conduct and compatibility with solid insulation. Even though research investigations endorse the use of these liquids as a substitute for MOs, there are numerous issues and challenges that must be explored. These research gaps and future research directions that must be targeted to enhance the understanding of ester fluids are presented below.

#### 6.2.1. Real-Time Condition Monitoring and Application in Existing Units

Several facilities around the globe are previously applying NE liquids in oil-immersed HV equipment. Longer-term in-service experience details regarding the condition monitoring of NE-immersed transformer may be valuable. These actual statistics on the insulation profile would be instrumental in determining the constraints of prospective aging makers. This knowledge may likewise be beneficial in knowing and establishing investigative instruments for NE loaded equipment. The mixability of NEs with MOs was demonstrated, and inclusion of NEs enhances dielectric conduct of MOs: mixture therefore developed is superior to MO dielectric fluids. Therefore, further research on retrofilling of NE liquids may be revealing if services intended to retrofill presently MOs filled units. The performance of MO-loaded (impregnated and aged) cellulose insulation arrangements when retrofilled with NE liquids should be revealed. Actual condition supervision data and retrofilling research may result in greater usage of NE liquids for oil-loaded equipment.

#### 6.2.2. Application in On–Load Tap Changers (OLTCs)

The frequency of failure in tap changers is higher than failures in transformers. The use of ester liquids in OLTCs is a fascinating challenge for subsequent research. Nevertheless, very few inquiries have stated on this subject. DGA studies of ester filled tap changers have been described. The deterioration of insulating liquids in tap changer contrasts from that of in the tank because of prompt variation in the potential of active terminals in the tap changers. Therefore, it is required to study the deterioration profile of oil in the OLTCs must be studied. With repeated undesirable releases and spark in tap changers, the degradation of oil is accelerated because of consistent electrical stressing. The effect of low-energy and high-energy exhausts on the operational performance of NE liquids in deficiency of solid insulation must furthermore be explored. Carbon precipitates accumulate on tap changer terminations, resulting in enhanced contact resistance, which further increases heating and carbon accumulation on lively components. This problem should be superior realized to assure the efficient function of ester liquids in tap changers.

#### 6.2.3. Streamer Propagation Analysis

Most of the studies on the pre-breakdown phenomenon have been conducted with point plane electrodes, whereas very limited studies have focused on sphere plane electrodes. Most of these investigations concentrated on studying streamer conduct and characteristics comprising streamer swiftness, streamer extent, streamer profile, charge and current. The impact of aging parameters and additional investigative/functional factors of streamer development has not until now been explored. Numerous studies have stated the influence of humidity and metal particulates on the breakdown phenomenon [117]. Nevertheless, cellulose particulates and other decomposition shreds also have a critical effect in real-time environment. The impact of electrode configuration (shape, gap span, tip radius) has been stated by multiple researchers, however excluding examining the aging of liquid and cellulose insulation [118].

#### 6.2.4. Comparison of Applicability Performance of Different Insulating Fluids

The functional applicability of various insulating fluids is an additional field of research. Few noteworthy explanations regarding to the applicability of different insulating fluids are outlined in Table 19.

#### 6.2.5. Additives and Chemical Scavengers

Natural esters indicate low LI resistance, so numerous investigators have made efforts to enhance their LI resistance by applying various additives [119]. Developed additives need comprehensive research to know their congruity with insulating liquids in working conditions. Thus, there is huge breadth for research on additions and chemical avengers to enhance the conduct of insulating liquids. Furthermore, opposition to oxidation and larger viscosity are key issues regarding NEs that muse be examined [120]. The additives should be developed for natural esters to enhance the cold flow characteristics, inhibition of oxidation during storage and to augment the material application compatibility, etc.

#### 6.2.6. End-of-Life Criteria Studies

A lifespan criterion for various dielectric liquids is an additional momentous issue for the industrial sector. The deterioration of NE liquids indicates nonexistence of colloidal elements at small and moderate aging [121]; conversely, solvable particulates are observed with the aging of NE liquids. There is still a huge opportunity for exploration of additives and absorbents that are coherent with different insulating liquids. The impacts of oxidized fluid on stability, reliability and performance of electric equipment need to be further investigated.

#### 6.2.7. Nanotechnology and Insulating Fluids

The application of application of nanotechnology is to augment the conduct of insulating liquids is another stimulating latest subject of research [122]. It had been stated that smart fluids formed with few particular nanoparticles (NPs) that smart fluids manufactured with some explicit nanoparticles (NPs) may be adapted to the preference of applications [123,124,125,126]. Numerous investigators studied BDS, viscosity, pour point, thermal features and streamer development in nano-based ester liquids [26,127,128]. A substantial enhancement is observed with the suspension of various types of NPs. However, future research must focus on stability, aging performance and compatibility of these nanofluids (NFs) with other transformer constituents [129]. Furthermore, the service performance of NFs and the impact of different suspension in natural esters are further required to be evaluated.

#### 6.2.8. Cost and Other Related Issues

More research efforts are compulsory to find ways to decrease the fabrication price, manufacturing lower price feedstock’s and recognize prospective marketplaces to balance price and accessibility. Persistent equipment stability, reliability and performance in a diversity of transformer categories, sizes, and ratings essential to be established to uplift manufacturer and customer trust. Environmental benefits and gains presented by this naturally renewable and environmentally friendly (natural esters) fluids over conventional fluids need to be acknowledged, advertised and promoted.

## 7. Challenges of Natural-Ester Insulating Fluids

Despite their attractive properties, natural esters are still in the evolving stage at the time for most practical applications. Market and governing pressures are rising to curtail the issues and challenges associated with the application of renewable and environmentally friendly insulating fluids to avoid the environmental hazards connected with the application of MOs in HV equipment. Furthermore, there are huge requirements to enhance effectiveness and approve more eco-friendly opportunities in electric networks; hence HV industry has been looking for superior alternatives and novel concepts. The challenges associated with the application and performance improvement of natural esters as insulating liquid are summarized below:

### 7.1. Base Fluid Selection

Natural esters generally extracted from seeds, which have two primary constituents, the oil portion and the solid segment possessing protein usually named as meal portion. This oil is derived from the crude base through technique specified as RDB, which signifies ‘Refined’, ‘Bleached’ and ‘Deodorized’. The practically accessible RDB class is a startup substance. To obtain oily portion, hydrocarbon solvents are applied and subsequently, they are eliminated. It is succeeded by cleansing and bleaching that includes processing by absorbent clay and screening. In deodorization operation, overheated steam is utilized to exclude stink inducing volatiles. Moreover, winterization operation is applied to eliminate readily subzero saturate fats [8]. The selection of appropriate base material for natural esters production is a huge challenge and sensitive process which may impact its characteristics; therefore, focused research work is necessary for this area to obtain a fluid that can be applied as excellent liquid insulation for the HV apparatus.

### 7.2. Production and Usage of Vegetable Oil

The production and application of natural esters in HV equipment to are enormous challenges to realize the reported positives. The researchers have acknowledged that natural esters need additional developments in their fabrication process to be applied in HV equipment. The oxidation stability of these fluids mainly depends on the fatty distribution, distillation process and the presence of natural antioxidants. The likely formation of natural esters obstructs their practical applications due to weak oxidation stability. Generally, liquid insulation in transformers stays in a unit for several years (almost 30–40 years if not exchanged in the midst). Natural esters naturally possess certain constituents which deteriorate in reasonably in limited time. More research is required in the production and application field of natural esters to take full advantage of their features.

### 7.3. Stabilization of the Oil

Natural esters in their natural profile lack adequate oxidative stability. Small oxidative stability implies, if unprocessed, the fluid would oxidize rapidly while usage, turning thick and polymerizing to a plastic-like consistency. Natural esters oxidation is a complex drastic chain reaction when oxygen breaks down fatty acids to develop volatile compounds. The degree of oxidation is influenced by light, heat, the fatty acid profile and the number of antioxidants in the oil. Natural esters have a tendency to deteriorate when air oxidation takes place because of intrinsic unsaturation. Natural esters loaded with polyunsaturated fatty acid are higher susceptible to oxidation than oils which are plentiful in monounsaturated fatty acids. Oils possessing more unsaturated fatty acids are oxidized more rapidly than the oils possessing less unsaturated fatty acids. As the amount of unsaturation rises, the amount of oxidation surges, making more complex combinations of hydroperoxides. Chemical modification of VOs and/or the application of antioxidants could be helpful to resolve the issue, however raise the cost. Chemical transformations may include fractional hydrogenation of the VOs and a shifting of their fatty acids. The challenge with hydrogenation is to evaluate at which point, the procedure is to conclude. Complete hydrogenation of fluid may result in solid derivatives like margarine. Depending on the required liquidity and pour point of the oil, optimal hydrogenation is specified.

All the insulating fluids must be exposed to an oxidation test. Generally, transformers have hermetic construction to avoid the oxidation however, leakage and periodic repair processes possibly will reveal fluid to the atmosphere. Over multiple years, fluid may deteriorate due to the oxidation process. Oxidation instability of natural esters is one of the biggest research challenges that obstruct its practical applications; therefore, more research work is required to look for ways and methods to enhance the stability of natural esters.

### 7.4. Improving Pour Point

Another challenge associated with natural esters is their higher pour point (the temperature upon which fluid absolves fluidity and does not pour/flow). Natural esters tend to frost at elevated temperatures in comparison to MOs. The freezing temperature is different for every oil due to the dissimilar configuration of each fluid. The great saturate amount in fluid may augment the freezing point. Unsaturated fluids generally possess pour point in the spectrum of −10 to −20 °C. This challenge may be partly handled by the use of chemical additives (pour point suppressants), winterization and/or merging with other liquids having smaller pour points. Different synthetic oils may be applied to this objective. The pour point could be improved by the above-mentioned process, but still, it requires huge research work to find out ways and methods to improve the pour point to use the insulating fluids efficiently in cold climates.

### 7.5. Reducing the Viscosity

**Figure 6:**
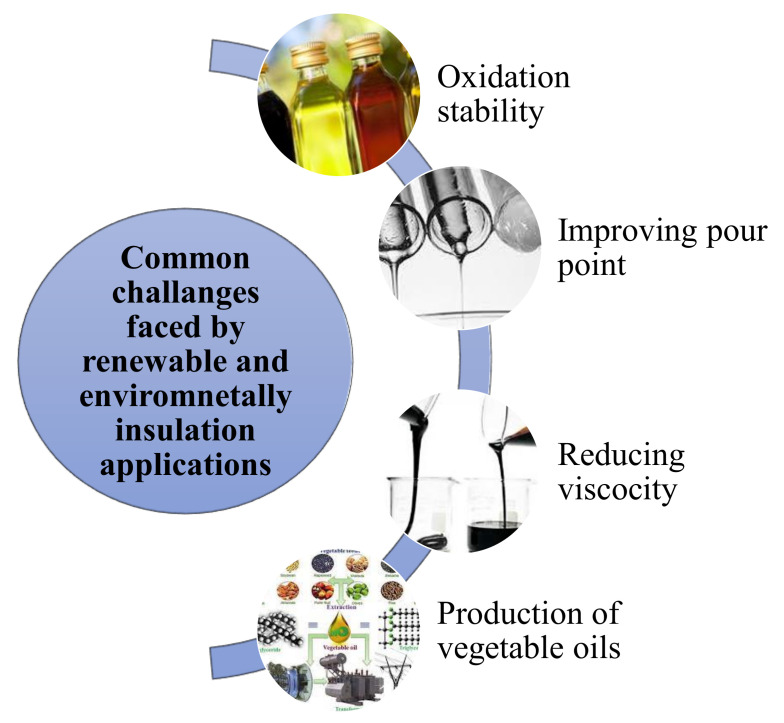
Common challenges related to environmentally friendly insulation fluids for HV applications.

The aging of natural esters fluids in the existence of air results in oxidation which further lead to an increase in viscosity. Thus, it is significant to be careful when filling the insulating fluid in transformers to eliminate oxygen. Researchers are making efforts to bring the viscosity of natural esters to the recommended level by applying various chemical modifications. The information on the viscosity of a fluid is necessary for the design of heat transfer equipment. The viscosity of natural esters may be decreased by the transesterification process. Transesterification is the process of exchanging organic group R” of an ester by organic group R’ of alcohol. Furthermore, few other processes to decrease the viscosity of VOs are dilution, microemulsion, pyrolysis and catalytic cracking [130,131]. It is highly needed to reduce the viscosity of the insulating fluid to design equipment that may efficiently transfer heat. The common challenges related to environmentally friendly insulation fluids for HV applications are outlined in Figure 6.

## 8. Potential Applications of Renewable and Eco-Friendly Insulating Fluids

Electric grids are globally growing; therefore, electrical equipment with HV designs, effective technologies and sustainable characteristics are highly desirable. Natural esters emerged as consistent, safer and green substitute liquids for HV equipment. Distribution transformers have been the primary preference to apply eco-liquids, substituting classical petroleum-based MO. The initial eco-friendly liquid filled transformers presented an outstanding demonstration and, founded on the encouraging implementation. Subsequently, eco-friendly liquids were deliberated for HV transformers. One of the main ester-immersed power transformers were developed in 2003 [132]. Different from distribution transformers, HV transformers are bigger apparatus which include a considerable bulk of insulating liquids and the huge amount of substances, predominantly copper, steel and solid-based constituents, for example paper, pressboard and layered high-density slabs, etc. Table 20 outlines the up-to-date distribution of applications of dielectric fluids in HV equipment.

High voltage equipment needs dielectric materials that have good insulating and cooling properties as well as good thermal stability and are readily biodegradable. Renewable alternative formulations with superior engineering or environmental properties are growing in popularity as these suitable candidates may offer superior insulating and cooling properties with good fire safety, nontoxicity and environmental friendliness profiles and they are being extensively acknowledged as prospect substitute for insulating fluids in prospect dielectric community. Natural esters possess better chemical and thermal stability. They offer pragmatic installation and supervisory benefits in multiple sensitive areas. Thermal deterioration of solid insulation reduces significantly in natural ester than deterioration in MO, which makes them a suitable applicant for transformers. Natural esters oxidize contrarily than MOs. The derivatives of oxidation don’t develop sludge sediments. Rather, fluid initiates to congeal and eventually polymerize. It will lead to an intense rise in viscosity which may enhance and develop the basis of a smart healing scheme for cable uses.

Eco-friendly liquids are different in physical, electrical, chemical and thermal characteristics than MOs; consequently, certain design, engineering and functional contemplations should be taken care of when they are implemented in power transformers [52]. For instance, both synthetic and natural esters have a greater viscosity than MOs which suggests they are less effective during heat transfer [133]. As a result, thermal design modifications should be mandatory because of the augmentation of top oil, winding and core temperatures. One-way to do this is by enhancing the size of oil conduits in coils [59]. Thermal modeling of windings and core are greatly suggested to gauge variance in temperature increase for NE-loaded transformer [134]. Alternatively, ester-based liquids possess greater thermal conductivity and to some extent larger heat capacity than MO that marginally compensate the adverse effect of its greater viscosity.

Electric equipment producers must encounter few electric challenges to implement eco-friendly ester liquids in HV applications, generally correlated with dielectric conduct of ester liquids under huge oil gaps [135]. The environmentally friendly liquids should have AC and switching and lightning impulse withstand voltages equivalent or higher to MO. During recent years, huge research has concentrated on studying the insulating effectiveness of ester-based liquids to acquire an understanding for suitable application of these liquids in HV applications. It has been revealed that natural esters indicate almost comparable streamer inception voltages with MO; though, the commencement of streamer in NE liquids spreads quicker and further, with additional offshoots than in MO [136]. Conversely, LI BDVs of ester liquids may be lower than MO at huge gaps [136]. Thus, broader insulating obstructions are usually deliberated when eco-friendly is applied in HV transformers. Alternatively, different from MO, ester-based liquids have permittivity values nearly to paper and pressboard insulation that shows superior stress dispersal in insulation arrangement (liquid–solid) in HV transformers. This electrical stress is inversely proportional to permittivity, so the configurations with greater permittivity carry lesser intensities of stress [137]. The dielectric conduct of ester-permeated cellulose is likewise good or even superior to when permeated with MO that may be ascribed to closer permittivity match, particularly efficient for smaller spaces [137]. Additional related feature in the development of HV transformers is associated with permeation procedure of insulating elements (paper, pressboard, etc.). Normally, a huge power transformer is consisting of loads of cellulose insulation, which should be permeated with liquids with a view to improving DS. Eco-liquids possess larger viscosity than MO, so the impregnation process consumes additional time, primarily in densely layered pressboard blocks. Nevertheless, this had been effectively revealed that an efficient mode to expedite this impregnation process and make it equal to the traditional process for MO, temperature for ester liquids may be raised [137].

Ester liquids have outstanding features useful for extension of transformer lifespan, which is mainly due to extensively slower aging of cellulose-based insulating paper in natural esters than MO under similar thermal stress [138]. This performance is partial because of the greater ability of ester liquids to captivate more humidity than MO. Moreover, there are certain indications that free acids emerged by the hydrolysis process may chemically react with solid, evolving reinforced and better substance [139]. There has been great progress concerning the use of ester liquids in huge transformers [139]. The evolution of NE power transformers has been carried out nearly at a similar time, with the bulk of natural ester ventures in the USA and Brazil, whereas synthetic ester transformers mostly mounted in Europe [120].

Since the last decade, natural esters have been applied in power transformers. Over and above 200 smaller and medium power transformers have been using natural ester liquid insulation [132]. Design novelties and process adaptations are effectively applied by transformer producers to meet greater voltages scales, up to 245 kV [139]. Subsequently, multiple studies were executed to formulate novel technologies to present NE in even greater voltage units. In 2013, the world’s largest power transformer (˃245 kV) packed with natural ester was effectively investigated. There is a 240 kV and 300 MVA vegetable oil-loaded extra HV power transformer formulated and manufactured by Siemens [120]. Performance outcomes of this unit have revealed that it behaves normally, producing completely no problem and functioning according to the requisite anticipations and standards.

Synthetic esters had been effectively applied for voltages up to 66 kV for almost 30 years. During the previous few years, synthetic esters, along with NEs, have been likewise deliberated for applications in power transformers. In 2004, an initial 238 kV and 135 MVA HV transformer occupied by synthetic ester were effectively established and mounted in Sweden [120]. Recently, an extra HV transformer up to 433 kV, filled with synthetic ester, has been developed [140].

**Figure 7:**
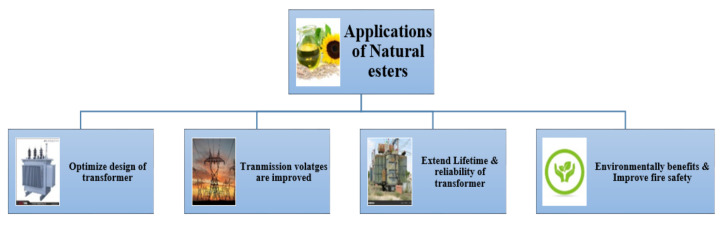
Overview of gains achieved by the application of natural esters as a substitute of MO.

Additional use of eco-friendly liquids is retrofilling of transformers, i.e., substituting the present service-aged MO with VO. This process has been conducted for approximately the last two decades, primarily in smaller units; although, this practice has become quite widespread in HV transformers up to 238 kV [141]. Lately, a scientific resolution for retrofilling HV single-phase transformer of 420 kV and 200 MVA was stated, by substituting its MO with natural ester [142]. For this application, it is highly significant to consider that retrofilled transformer would have a blend of liquids, synthetic and NE with small loading of remaining MO. Ester fluids present ample miscibility with MO; on the other hand, if the MO loading is larger than 8%, fire opposition and biodegradability features of the eco-friendly liquids may be reduced. The overview of advantages attained by the application of natural esters as substitute of MO is briefly stated in Figure 7.

## 9. New and Innovative Technologies for Environmentally Friendly Fluids

Novel R & D exertions have been directed in the last few years to enhance the conduct of environmentally friendly liquids for HV applications. Innovative substitutes of seed oils are deliberated to formulate the latest varieties of NE liquids; moreover, state-of-the-art engineering progression and chemical modification of the VO configuration has also been deliberated. Alternatively, nanotechnology has evolved as a technological solution to improve natural ester liquids for HV equipment.

### 9.1. New Vegetable Oil Formulations

NE insulating liquid developments is generally founded on soybean, canola, rapeseed, and sunflower oils. Lately, novel seed oils have been investigated and suggested as a substitute to formulate novel natural insulating liquids with enhanced features. Kumar et al. concluded that rice bran-based liquids indicate appropriate features such as BDV, flash point, fire point and resistivity; however thermal and other electrical characteristics are required to be improved. Palm oil has been deliberated as liquid insulation for transformers, specifically in certain states of Asia and Africa [143]. Abdelmalik made efforts to enhance the oxidation stability of palm kernel oil by chemical adjustment through the esterification reaction. The manufactured ester must possess appropriate BDS and physicochemical characteristics to be a successful candidate for liquid insulation for HV transformers [144]. In 2013, Azis et al. revealed that palm fatty acid manifests the highest value of BDV than other tested vegetable oils [145]. Another similar study concluded that RDB palm oil indicates superior dielectric characteristics than MO [146].

In recent years, nonedible oils have emerged as innovative substitutes for vegetable oils for transformers. A popular instance of non-edible oilseeds is *Pongamia pinnata* that is a middle-sized evergreen tree. Masarakall et al. concluded that the characteristics of *Pongamia pinnata* liquid may be enhanced by the esterification method and by using additives for pour point and oxidation stability; consequently, it may be regarded as the dielectric liquid for transformers [147]. Alternatively, Mariprasath et al. mentioned that Pongamia pinnate-based liquids indicate superior BDV than MO and adequate viscosity to be applied liquid insulation for transformers [148].

Jatropha curcas plant is an additional nonedible substitute that may be used for liquid insulation. This plant is usually used in conventional medicines, soap development, fuel for stoves, etc. [149]. In 2014, Sitorus et al. investigated electrical and physicochemical features of Jatropha curcas methyl ester oil (JMEO), concluding that JMEO fulfills conditions to be applied as an eco-friendly substitute for HV transformers, apart from its worst flash point. It was also concluded that the streamer formation and its stopping length of JMEO are analogous to MO [150]. In 2017, Evangelista et al. described the formulation of new liquid insulation for HV applications based on Jatropha curcas oil, which manifested appropriate characteristics. Nevertheless, the author revealed the need for the incorporation of antioxidants to avoid its oxidation at rising temperatures [151]. In the same way, Beltran et al. described a novel filtering method to attain Jatropha curcas insulating liquid that indicates suitable conduct to be applied in HV equipment. Nevertheless, problems of great pour point and small oxidation stability may be enhanced by additives [152].

Castor oil (*Ricinus communis*) is also a plant that has been deliberated as a non-edible preference for liquid insulation applications. Adeolu et al. stated that raw castor oil is not appropriate to be applied for HV applications since its BDVs are less than the lowest necessary for the application [153]. However, Naranpanawa et al. (2013) concluded that castor oil has suitable BDV however, also presents an exceptional pour point with the only associated shortcoming of large viscosity [154]. Lately, Banumathi et al. studied that castor oil indicates greater BDV and good fire point than MO. At the same time, the kinematic viscosity of this oil was noted greater than MO [155].

### 9.2. Nanofluids Based on Natural Esters

During the previous couple of decades, nanotechnology applications have displayed extraordinary development in numerous engineering and scientific areas [156], manufacturing advanced materials. A remarkable illustration of the advantages of nanotechnology is specified by the innovation of cutting-edge fluids based on nano ideas that are termed as nanofluids (NFs). The word “nanofluid” was offered by Choi et al. in 1995 and this is stated to as colloidal liquid consists of a liquid segment and nanoparticles (NPs) uniformly distributed [157]. NFs are deliberated as a substitute for the contemporary generation of liquid dielectric insulation for HV equipment [157]. Recently, it is one of the utmost dynamic research fields, and favorable findings to improve both heat transfer and insulating features of typical MO have been described [158,159,160]. Nanotechnology has been deliberated to enhance numerous features of renewable and eco-friendly liquid insulation for HV equipment. In reality, NFs are one of the most explored research fields concerning nanotechnology applications in the HV apparatus. One of the preliminary studies on NE-based NFs was accomplished by Li et al. which concluded that suspension of Fe_3_O_4_ NPs may enhance the LI BDVs by 12% [159]. In another recent study, Karthik et al. concluded that the suspension of SiO_2_ NPs may enhance the BDV of corn and coconut oil [161]. Du et al. developed a novel sort of NE-based NF prepared with boron nitride (BN) NPs that indicated superior heat transfer properties and enhanced AC BDV (34%) as compared with NE as the carrier oil, possessing slight loadings of NPs (0.1 wt%) [161].

Saenkhumwong et al. concluded that enhancement in BDV by the suspension of ZnO NPs into soybean and palm oil [162]. In another study, Srinivasan et al. concluded improvements in insulating, physical and thermal features of various kinds of NE liquids by the suspension of TiO_2_ and ZnO NPs [163]. Peppas et al. established an ultra-stable NE-based NF for HV applications that consisted of Fe_2_O_3_ nanocrystals. This developed eco-friendly NF is considered to be totally stable colloidal arrangement, even after 10 months; in addition, it exhibited outstanding DS as fresh and posted aging experiments [164].

## 10. New Emerging and Changing Trends

Urban migration globally changing the demography of cities because of populace density evolution; consequently, a surge in the extent of energy and delivery of consistent and effective electricity may be compulsory. The HV apparatus must be capable of coping with these dynamic and challenging conditions. Moreover, it must deliver outstanding efficiency however also should be secure and ecologically pleasant to be fitted and activated in metropolitan areas.

It is well acknowledged that MO is the utmost extensively used dielectric fluid in the HV apparatus, having occupied major market segments in 2016. Nevertheless, biodegradable dielectric liquids have been deliberated as a suitable alternative for MO because of their outstanding characteristics, fire resistance and environmental conduct. It is anticipated that the use of this ecologically welcoming liquid insulation for HV equipment would accelerate in the impending future. The applications of NEs have developed a growing trend and an expanding research area both in academia and manufacturing; consequently, the influx of more coolants based on NEs with improved features and lesser prices are projected. Enhancements in the cold performance and oxidation stability along with a decline in viscosity are leading subjects concerning studies in NEs in the HV apparatus. Moreover, non-traditional seed oils have been explored to formulate NEs liquids, containing edible and nonedible plants; therefore, novel VOs established on cutting-edge preparations would be projected to materialize in the prospect. In recent times, there has been a strong tendency to apply environmentally friendly liquid insulation at mounting voltages and power ratings; as a result, investigation in the electrical and thermal scheme, engineering deliberations, and monitoring and diagnosis in real time is compulsory to acquire the understanding that may be useful for the practical application of NEs in extra HV equipment. Alternatively, it is projected that a large quantity of service-aged transformers would be deliberated for retrofilling/substituting MO with environmentally friendly liquids because of multiple benefits, for example enhancing its fire security, its consistency and its rest of useful life.

Recently, NFs are a lively investigation subject; there are numerous studies that have revealed an enhanced efficiency over traditional dielectric liquids. NFs may signify an opportunity to reduce the dimension of impending transformers primarily due to enhanced heat transfer ability and greater dielectric strength. As a result, novel R&D ventures concerning NE-based NFs are projected in the following few years, in search of the formulation of greater stability and conduct NFs and their application in HV equipment [165].

In view of environmental hazards, fire safety, health vulnerability, call for footprint decline, insulating fluids based on NEs is the new-generation insulating fluids that are going to replace MOs. NEs score over MOs on ecological apprehensions with abundantly biodegradability, non-poisonous and are viable, ecological and sustainable origin with carbon-neutral traits. In addition to environmental favorability, natural esters are the insulating fluids that have characteristics crucial for HV equipment.

## 11. Conclusions

In view of environmental hazards, fire safety, health vulnerability, call for footprint decline, insulating fluids based on NEs are the next-era insulating fluids that will replace MOs. NEs score over MOs on ecological apprehensions with abundantly biodegradability, nontoxicity and are a viable, ecological and sustainable origin with carbon-neutral traits. In addition to environmental favorability, NEs are insulating fluids that have characteristics crucial for HV equipment. Natural esters prove as fire safe with greater fire point (“K” category fluid). Researchers have conducted multiple studies to apply natural esters as insulating fluid alternatives. The application of natural esters as insulating fluid may perform a critical role in aiding the world to decrease the ecological effect of MOs. The progress of natural esters fluids satisfies prevailing necessities for environmentally friendly insulating fluids. However, environmental gains are of excessive curiosity, in the prospect, when petroleum yields are ultimately going to come to an end, and there may be severe scarcities even by the middle of this century. Luckily, the preliminaries have previously been placed by the development of appropriate substitute insulating fluid. The potential applications for these natural ester fluids may include transformers, circuit breakers, cables, tap changers, capacitors and cables, etc. Moisture saturation limits of NEs being extraordinary as they may grasp extra moisture. Moderate oxidation stability of vegetable oils obliges superior attention and airtight structure of the container. NEs also manifest stray gassing trends and produce ethane and hydrogen—resulting in misconception in DGS evaluation. The serious issue of the space constraints of HV equipment (e.g., transformers) may be decreased by the application of high-temperature natural esters dielectric fluids.

Part 2 of the article argues merits, demerits and dielectric design challenges and determines the appropriateness of NEs both for retrofilling and innovative designs of HV equipment. Moreover, the investigation of natural-ester insulating fluids needs additional studies and experiments.

## Figures and Tables

**Table 1:** Properties of fluid insulation.

Electrical Properties	Chemical Properties	Physical Properties
Alternating current (AC) Breakdown strength (BDS)	Oxidation stability	Thermal conductivity
Lightning impulse (LI) BDS	Gassing features under electric stress	Specific heat
Partial discharge inception voltage (PDIV)	Ionization situations (silent discharge)	Coefficient of volume expansion
Dielectric dissipation factor	Gassing features under high-temperature pyrolysis conditions (thermal and disruptive discharge)	Viscosity
Volume resistivity	Neutralization value	density
Permittivity	Saponification value	Pour point
Contaminants	Sulfur staining and corrosion	Refractive index
	Nitrogen content	Molecular weight
	Ionic contamination	Solvent power
	Moisture content	Vapor pressure
		Flammability
		Interfacial tension

**Table 2:** Summary of characteristics of insulating fluids applied in HV equipment [5].

Features	MO	Silicone Fluid	Synthetic Ester	NE
Category	Filtered from crude petroleum	Synthetic	Synthetic	Refined vegetable oil
Major component	Composite combination of hydrocarbons	di-alkyl silicone polymer	Pentaerythritol tetra ester	Plant-based natural ester
Origin	Refined from oil	Prepared from chemicals	Developed from chemicals	Derived from crops
Biodegradability	Slowly biodegradation	Really slow to biodegradation	Readily biodegradable	Readily biodegradable
Oxidation stability	Good stability	Outstanding stability	Outstanding stability	Normally oxidation vulnerable
Moisture saturation at ambient (ppm)	55	220	2600	1100
Flash point, °C	160–170	˃300	˃250	˃300
Fire point, °C	170–180	˃350	˃300	˃350
Fire categorization	O	K	K	K

Note: O-flash point ˂ 300 °C, K-flash point ˃ 300 °C as per IEC 61100 standard VOs categorized as low-flammability liquids as per IEC standards.

**Table 3:** Electrical features of natural ester fluid and mineral oil (MO).

Electric Features	MO	NE Fluid	References
Dielectric strength (BDV), kV	54.9	56.7	[46]
Dielectric dissipation factor	0.081	0.45	
Specific resistance, 10^12^ ohm cm^─1^@80 °C	220	3	
Gassing tendency, μL/min	−5	−79	

**Table 4:** Comparison of alternating-current breakdown strengths (AC BDVs) for different insulating liquids.

Experiment	MO	Synthetic Ester	NE	Silicone Oil	Low Viscosity Silicone Oil	References
IEC 60,156 2.5 mm	70 kV	˃75 kV	˃75 kV	50 kV	70 kV	[50,51]
ASTM 1816 1 mm	–	–	37 kV	–	–	
ASTM 1816 2 mm	60 kV	–	76 kV	–	–	
ASTM D877	55 kV	43 kV	46 kV	43 kV	–	

– signifies not available data.

**Table 5:** Impulse BDV of various insulating fluids with spherical electrodes.

Gap Distance (mm)	Mean Stress (kV/mm)			Ref.
	MO	Silicone Oil	VO (MIDEL 7131)	
1.0	40.6	42.0	33.4	[50,51]
2.5	38.3	47.7	33.0	
2.0	45.8	42.3	44.6	
2.5	39.0	34.0	36.5	

**Table 6:** Evaluation of physical characteristics of natural esters (NEs) and MO.

Physical Characteristics		MO	NE liquid	Ref.
Viscosity, cSt	@40 °C	7.800	35.12	[46]
	@100 °C	2.240	8.010	
Density, @ 25 °C/Cg/cc		0.855	0.924	
Pour point, °C		−40	−21	
Flash point, °C		145	275	
Interfacial tension, mN/M		40	30	

**Table 7:** Relative density of various insulating fluids.

Feature	Standard	MO	NE Fluid	Reference
Relative density (g/mL) (15 °C/15 °C)	ASTM	≤ 0.91	≤ 0.92	[64]
	IEEE	≤ 0.91 at 15 °C/15 °C	≤ 0.96 at 25 °C	
Density at 20 °C (g/mL)	IEC	Max. 0.895	Max. 1.1	[65,66]

**Table 8:** Density measurements of different types of vegetable oils.

Temperature °C	Density, g/cc				Reference
	Soybean Oil	Coconut Oil	Corn Oil	Rapeseed Oil	
23.9	0.9193	–	0.9188	0.9078	[67,68]
37.8	0.9082	0.9107	0.9082	0.8977	
48.9	0.9023	0.9033	0.9028	0.8829	
60.0	0.8939	0.8949	0.8939	0.8829	
82.2	0.8795	0.8795	0.8800	0.8681	
100.0	0.8674	0.8669	0.8679	0.8564	
110.0	0.8615	0.8695	0.8610	0.8501	
25.0	0.915–0.918	0.916–0.918	0.915–0.917	–	

– signifies not available data.

**Table 9:** Pour points of various insulating fluids.

Features	Standard	MO	NE Fluid	Reference
Pour point (°C)	ASTM	≤ −40 °C	≤ −10 °C	[80]
	IEEE	−	≤ −10 °C	
Tendency to develop voids		Natural esters presented a reduction in trend to form cavities after cooled further than its pour point temperature		[80,81]
Cold launch/start		No specific care is necessary while cold launch of NE filled transformer		[81]
Ambient falls below 0 °C		Endorsed to operate transformer under without load circumstance		[82]

– signifies not available data.

**Table 10:** Fire and flash point particulars of dielectric fluids.

Characteristics	Standard	MO	NE	References
Fire protection category	IEC	O1	K2	[65,66,80]
Flash point (°C)	ASTM	Limit 145	Limit 275	
	IEEE	−	Min. 275	
	IEC	≥135	Min. 250	
Fire point (°C)	ASTM	Limit 170	Limit 3000	
	IEEE	−	Min. 300	
	IEC	−	Min. 300	
Calorific value MJ/kg	−	46	37.5	
Transformer parting space obligation from other transformer or structure or another substation apparatus	FM Universal Standard3990	1/10^th^ clearance essential for MO filled transformers		
Condition of the fire extinguishing system		required	Not essential even for indoor applications	

− signifies not available data.

**Table 11:** Fire and flash points of various dielectric fluids.

Fluid	Flash Point (°C)	Fire Point (°C)	Category	References
MO	160−170	170–180	O	[5]
Silicone fluid	˃300	˃350	K3	
Low viscosity silicon fluid	268	312	K3	
Synthetic ester	˃250	˃300	K3	
NE	˃300	˃350	K2	

**Table 12:** Relative evaluation of extraordinary fire point and smaller inflammable fluid immersed transformer categories and their traits.

Traits	MO	Hydrocarbon Oil	Silicone Oil	NE	References
Fire opposition	Poor	Outstanding	Outstanding	Outstanding	[50,51]
Environmental impression	Moderate	Reasonable	Reasonable	Outstanding	
Life probability at max. temperature rating	Good	Good	Good	High	
Efficiency	High	High	High	High	
Sound level	Low	Low	Low	Low	
Functioning temperature	Low	Low	Low	Low	
Impurity resistance	Outstanding	Outstanding	Outstanding	Outstanding	
Overload capability	Good	Outstanding	−	Outstanding	
Initial cost	Low	Low/reasonable	High	Reasonable	
Energy costs	Low	Low	Low	Low	
Recycle/discarding costs	Low	Low	High	Low	

− signifies not available data.

**Table 13:** Water solubility of different insulating fluids.

Fluid	Ester Associations	Approx. Moisture Saturation at 23 °C (ppm)	Reference
MO	0	55	[50,51]
Silicone oil	0	220	
NE	3	1100	
Synthetic ester	4	2600	

**Table 14:** Chemical characteristics of different insulating liquids.

Experiment Factor	Parameters	MO	NE	References
Moisture level, mg/kg	˂200	15.0	20.7	[46]
Neutralization value, mg of KOH/g of oil	˂0.06	0.01	0.08	
Erosive sulfur	Non-erosive	Non-erosive	Non-erosive	

**Table 15:** Oxidization stability limits for various insulating fluids.

Oxidization Stability Limits	Mineral Oil	Natural Ester	Reference
According to IEC, experiment procedure is similar for both fluids, except a period of expedited aging	164 h at 120 °C	48 h at 120 °C	[74]
Oxidation stability	MO ˃˃˃ NE		[80]
Oxidation stability values attained by Rotating Bomb Oxidation test (RBOT)	300 min	˂40 min	

**Table 16:** Dissolved gas analysis (DGA) exploration methods of dielectric fluids.

Method of Measurement	MO	NE Fluid	Reference
DGA method	Identical analysis techniques are appropriate for NEs; though amidst all Duval triangle is the utmost trustworthy procedure to be used for NEs		[82,95,96]
Duval triangle for electrical fault	Electric defects in NEs fluid (electric discharges, breakdown and PDs) may be discovered by the present MO dual triangle without any change.		[82,95]
Duval triangle for thermal fault	Thermal faults may be discovered for NEs by changing zone limitations for thermal defects T1, T2 and T3 of present MO Duval triangle.		
Relevant Duval triangle for small thermal issues and stray gassing	Duval Triangle 4	Duval Triangle 6	[29,82]

**Table 17:** Environmental characteristics of various insulating fluids.

Characteristics	MO	Synthetic Ester (SE)	NE	References
Biochemical oxygen demand 5-day SM5210B (ppm)	6	24	250	[12,102,103]
Biodegradability				
21-day CEL-L-33	˂30%	80%	97% to 99%	
OECD 301 classification	Not biodegradable	Readily biodegradable	Readily biodegradable	
IEC 61039 grouping	Not biodegradable	Fully biodegradable	Fully biodegradable	
Toxicity	Yes	Low	No	
Sustainability	No	Yes	Yes	
Fire threat evaluation category (IEC 61039)	O (fire point 110–185 °C*)*	K (fire point ˃ 300 °C*)*	K (fire point ˃ 300 °C*)*	
Emission profile	Unacceptable	Questionable	Acceptable	

**Table 18:** Properties of transformer fluids (typical values/limits) [5].

Properties	Vegetable Oil	High temperature MO	Silicone Fluid
Appearance	Light yellow ^a^	Light yellow	Colorless
Specific gravity (25 °C)	0.91–0.92	0.89	0.96
Kinematic viscosity (cSt) 0 °C 25 °C 40 °C 100 °C	170–250 55–75 33–45 8–10	2200 300 125 13	95 50 38 16
Pour point, °C	−15 to −25	−20 max.	−50 max.
Interfacial tension (IFT), dynes/cm	25	40–45	25
Flash point, °C	310–325	275 min.	300 min.
Fire point, °C	354–360	160–180	340
Moisture content, ppm dry oil	50–100	10–25 ^b^	50
(water solubility at 25 °C)	1200	60	200
Thermal constants Heat capacity, cal/g °C	0.50–0.57	0.488	0.363
Thermal conductivity, W/mK	0.17 ^a^	0.13	0.15
Coefficient of expansion/°C	0.0007	0.00073	0.00104
Chemical Chemical type	Ester 0.06 ^a^ Pass	Hydrocarbon 0.01 Pass	Organo-silicon 0.01 Pass
Electrical Dielectric constant at 20 °C Volume resistivity at 25 °C, Ohm cm	3.1 10^14^	2.2 10^14^–10^15^	2.71 10^14^
Breakdown voltage, kV ASTM D 1816, 2-mm gap electrodes	74 ^a^	60	–
Impulse breakdown voltage, kV (needle negative)	116 ^a^	145	136
Dissipation factor (%) 25 °C 100 °C	0.25 ^a^ 1.00 ^a^	0.05 max. 0.3 max.	−0.01 –
Grassing tendency-ASTM D2300	−50 ^a^	−19 to 20	N/A
Biodegradability CEC-L-33 (21 days)	97–99	30	Very low

^a^ For BIOTEMP fluid; ^b^ Varies with transformer rating.

**Table 19:** Workability observations for various insulating fluids.

Characteristics	MO	Synthetic Ester	NE	References
Mixability	–	Miscible in all percentages	Miscible in all percentages	[12,102,103]
Appropriateness for cold environments	Acceptable	Acceptable	Questionable	
Soluble particles with aging	Yes	Yes (with bigger aging)	Yes (with bigger aging)	
Colloidal particles with aging	Yes	No (with lower/moderate aging)	No (with lower/moderate aging)	
Major gases created post aging	H_2_ and C_2_H_2_	CO and CO_2_	CO and CO_2_	
Oxidation stability	Acceptable	Acceptable	Questionable	
Absorption capacity	Rises briskly with aging	Rises moderately	Initially high	
Antioxidants	required	Required	Strongly required	
Gelling	No	No	Partially yes (for breathing units)	
Gelling	faster	slower	slower	

− signifies not available data.

**Table 20:** Use of different insulating fluids in transformers.

Apparatus	MO	Silicone Fluid	Synthetic Ester	Vegetable Oil (Natural Ester)	References
Power transformers	A	X	B	B	[50,51]
Traction transformer	A	A	A	X	
Distribution transformer	A	A	A	A	
Instrument transformer	A	X	X	X	

(A = mostly used; B = less frequently used; X = presently not used).
